# Defining orthoplastic limb salvage centers: a systematic review

**DOI:** 10.1007/s00402-026-06325-0

**Published:** 2026-05-02

**Authors:** Omar Moussa, Fernando J. Pacheco, Floris V. Raasveld, Marcos R. Gonzalez, Kamilcan Oflazoglu, Marco J. P. F. Ritt, Ian L. Valerio, Hinne Rakhorst, Krystle R. Tuaño, Kyle R. Eberlin

**Affiliations:** 1 Massachusetts General Hospital, Harvard Medical School, Boston, USA; 2 Amsterdam University Medical Center, Amsterdam, Netherlands; 3 Harvard Medical School, Boston, USA; 4 Department of Plastic, Reconstructive and Hand Surgery, Rode Kruis Ziekenhuis, Beverwijk, Netherlands; 5 Department of Plastic, Reconstructive and Hand Surgery, Erasmus Medical Center, Rotterdam, Netherlands; 6 Department of Plastic, Reconstructive and Hand Surgery, University of Groningen, Groningen, Netherlands

**Keywords:** Reconstruction, Limb salvage centers, Orthoplastic surgery, Systematic review, Organizational framework, Quality improvement, Healthcare management

## Abstract

**Introduction:**

Limb salvage centers have increased in number over time, but lack standardized defining criteria. This systematic review aimed to assess organizational features of limb salvage centers and determine whether orthoplastic centers, in comparison to vascular limb salvage centers, represent a distinct care model that may benefit from standardization.

**Methods:**

We conducted a systematic review of publications related to limb salvage centers by searching MEDLINE, Embase, Web of Science, and Cochrane databases from their inception through 2024. We quantified binary data extraction as a reporting score of 26 organizational features across six structural care domains for limb salvage centers, based on a validated quality measurement framework. Organizational features differentiating distinct center types were identified to establish a quality framework for orthoplastic centers. Statistical comparisons between center types were performed using appropriate tests (*p* < 0.05).

**Results:**

Of 118 included studies, orthoplastic (*n* = 43) and vascular (*n* = 48) centers represented 77% of all studies. Recent increases in orthoplastic publications show substantial variability in organizational features. Orthoplastic center literature more frequently reported plastic surgery consultation criteria (*p* < 0.001), surgical outcomes (*p* < 0.001), and centralized network integration (*p* ≤ 0.006), highlighting acute reconstructive approaches. Vascular center studies documented significantly more organizational team features (*p* < 0.001) and quality systems (*p* = 0.033), reflecting established care frameworks for chronic disease management. Six organizational features characterized orthoplastic centers with > 70% prevalence, providing a benchmark framework with standardization priorities.

**Conclusion:**

Orthoplastic limb salvage centers demonstrate distinct care paradigms that benefit from standardization. Our findings suggest structural benchmarks to support the need for standardized development of orthoplastic limb salvage centers.

**Supplementary Information:**

The online version contains supplementary material available at 10.1007/s00402-026-06325-0.

## Introduction

Limb salvage is a priority in patients following severe trauma, oncologic resection, or vascular compromise [1–5]. These procedures require coordinated multidisciplinary care to optimize functional outcomes and minimize complications [6, 7]. Limb salvage centers have evolved using primarily vascular or orthoplastic approaches, with each approach having unique patient populations, care pathways, and organizational structures [8, 9].

Vascular limb salvage centers primarily treat chronic limb-threatening ischemia and diabetic complications, guided by established frameworks [10]. In contrast, orthoplastic centers often emphasize acute trauma reconstruction, combining orthopaedic and plastic surgery expertise [11, 12]. Despite orthoplastic approaches showing well-documented clinical benefits, no standardized organizational frameworks for orthoplastic centers have been established [13, 14].

Organizational factors may impact patient outcomes in complex surgical care [15]. However, the specific organizational features that characterize different limb salvage center types remain unclear. The absence of accepted standards has led to the development and practice of orthoplastic centers without a standardized framework. This situation may result in practice variations across orthoplastic centers, potentially impacting limb salvage outcomes [16–19]. As a result, there is a need to better define “orthoplastic centers”.

This study aimed to assess the structural and operational characteristics of limb salvage centers based on an established healthcare quality measurement framework. We sought to quantify organizational features that differentiate orthoplastic centers from vascular centers and to provide evidence-based benchmarks in the standardization of limb salvage care.

## Methods

### Search strategy and study selection

This systematic review followed the Preferred Reporting Items for Systematic Reviews and Meta-Analyses (PRISMA) guidelines [20]. A comprehensive search was conducted across four databases: MEDLINE (via PubMed), Embase, Web of Science, and the Cochrane Library from their inceptions through December 2024 (Supplement 0).

Studies were included if they described organizational characteristics, structural features, care protocols, or healthcare delivery patterns of limb salvage centers. Studies were required to describe surgical limb salvage centers treating any limb-threatening condition (including trauma, oncologic, vascular, or infectious etiologies). Non-empirical studies, studies focusing solely on surgical techniques or lacking an organizational context, and non-English studies or those unavailable as full text were excluded.

We acknowledge that limb-threatening pathologies differ fundamentally in care requirements; accordingly, our primary analytic strategy stratified studies by center type, which broadly reflects the dominant clinical indication each center addresses (e.g., orthoplastic centers predominantly manage acute trauma and complex reconstruction, whereas vascular centers predominantly manage chronic limb-threatening ischemia).

### Data extraction and classification

Two reviewers independently conducted title and abstract screening using systematic keyword searches to identify organizational content. Full-text assessment was performed independently using predetermined criteria. Disagreements were resolved through discussion with a third reviewer.

We extracted study characteristics including publication details, geographic location, study design, population size, hospital type, trauma center designation, and center establishment year. Center types were classified as orthoplastic, vascular, orthopaedic, ortho-trauma, ortho-oncologic, or no specific designation (no designated center) based on author affiliations and stated center focus.

### Scoring framework

We developed a binary scoring system based on the Donabedian healthcare quality framework, a validated model that evaluates healthcare quality through structural, process, and outcome measures [10,21]. Each of 26 organizational features was scored as present (1) or absent (0) based on documentation in published studies. Features were grouped into 6 domains: Team Organization (3 features), Care Pathways (4 features), Care Delivery (4 features), Outcome Metrics (6 features), Quality and Safety (5 features), and Network Integration (4 features). Each study received a total score [ranging from 0 to 26] representing the sum of documented organizational features.

### Risk of bias assessment

Each study’s risk of bias was independently evaluated. The methodological index for non-randomized studies (MINORS) was used for non-randomized studies. Case series and case reports were assessed using the Joanna Briggs Institute (JBI) critical appraisal tools. Systematic reviews were evaluated using the ROBIS tool, while narrative reviews were assessed using the Scale for the Assessment of Narrative Review Articles (SANRA). Guidelines and consensus statements were evaluated using the AGREE II instrument.

### Statistical analysis

We performed descriptive statistics for all organizational features, calculating reporting frequencies and percentages for categorical variables and medians with interquartile ranges (IQR) for continuous variables. All statistical analyses were performed using Stata IC Version 17 (StataCorp LLC, College Station, Texas, USA). After appropriate parametric assessment, comparative analyses used Pearson’s chi-squared tests for binary variables, and Wilcoxon rank-sum tests or Kruskal-Wallis for continuous variables, depending on the number of centers compared. Statistical significance was set at *p* < 0.05.

## Results

### Study results and trends

**Fig. 1:**
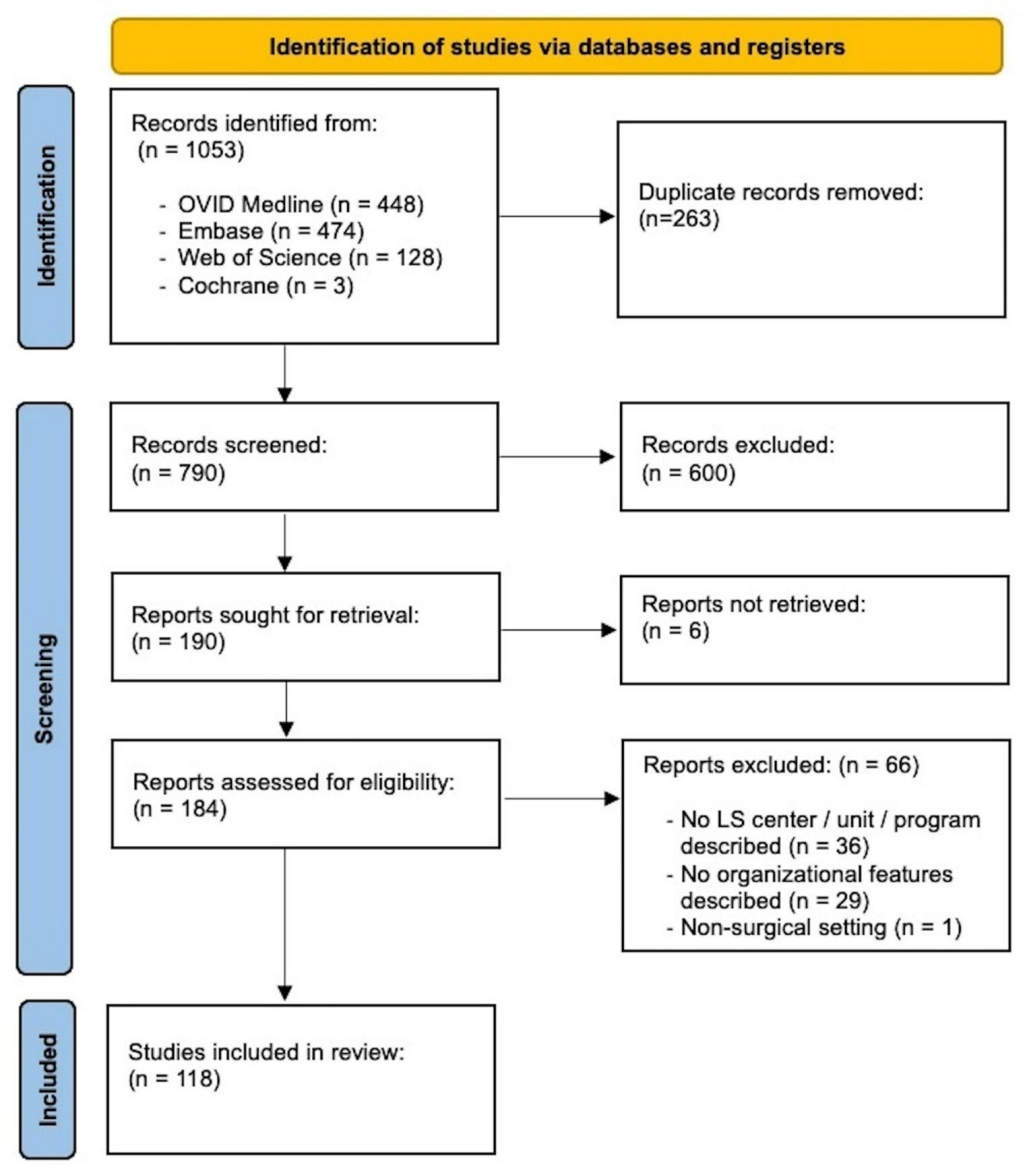
Flowchart. The flowchart illustrates the identification, screening, and inclusion of studies for the review, following the PRISMA (Preferred Reporting Items for Systematic Reviews and Meta-Analyses) guidelines. The process began with the identification of 1053 records from databases. After removing 263 duplicate records, 790 records were screened, and 600 were excluded due to irrelevance. Of the 190 reports sought for retrieval, 72 were not retrieved for reasons such as lack of description of a limb salvage center/unit/program (*n* = 36), absence of organizational features (*n* = 29), unavailability of full text (*n* = 6), or being in a non-surgical setting (*n* = 1). Ultimately, 118 studies met the eligibility criteria and were included in the review.

Among 1053 articles that were identified, 118 articles met inclusion criteria and were included in this study (Fig. 1; Table 1; Supplement 1). The included studies primarily described two major center types: orthoplastic centers (*n* = 43, 36%) and vascular centers (*n* = 48, 41%), which together represented 77% of all studies. Additional center types included ortho-trauma (*n* = 12, 10%), ortho-oncologic (*n* = 8, 7%), orthopaedic (*n* = 3, 3%), and studies without a specified center designation (*n* = 4, 3%).

**Table 1:** Study and center characteristics by limb salvage center type

Factor	Total	No center	Ortho-oncologic	Ortho-trauma	Orthopaedic	Orthoplastic	Vascular
*N*	118	4	8	12	3	43	48
Publication year, median (IQR)	2020 (2015, 2022)	2018 (2014, 2022)	2018 (2015, 2020)	2020 (2017, 2021)	2009 (1999, 2017)	2021 (2015, 2022)	2020 (2017, 2022)
Program establishment year, median (IQR)	2011 (2006, 2017)	–	2002 (2002, 2002)	2016 (2013, 2017)	2007 (2007, 2007)	2011 (2008, 2014)	2011 (2005, 2018)
Population size, median (IQR)	162 (72, 597)	–	298 (48, 4874)	135 (35, 672)	53 (34, 86)	101 (65, 200)	406 (194, 871)
*Study design*							
Case series	5 (4%)	0 (0%)	1 (12%)	2 (17%)	0 (0%)	2 (5%)	0 (0%)
Cross-sectional	4 (4%)	0 (0%)	0 (0%)	0 (0%)	1 (33%)	3 (7%)	0 (0%)
Other	12 (10%)	1 (25%)	0 (0%)	0 (0%)	0 (0%)	1 (2%)	10 (21%)
Prospective cohort	10 (9%)	1 (25%)	1 (12%)	0 (0%)	0 (0%)	6 (14%)	2 (4%)
Retrospective cohort	74 (63%)	0 (0%)	6 (75%)	9 (75%)	2 (67%)	28 (65%)	29 (60%)
Review	13 (11%)	2 (50%)	0 (0%)	1 (8%)	0 (0%)	3 (7%)	7 (15%)
*Hospital type*							
Absent	6 (5%)	1 (25%)	1 (12%)	0 (0%)	0 (0%)	0 (0%)	4 (8%)
Academic	72 (62%)	2 (50%)	3 (38%)	8 (67%)	3 (100%)	29 (67%)	27 (56%)
Military	4 (4%)	1 (25%)	0 (0%)	1 (8%)	0 (0%)	0 (0%)	2 (4%)
Multiple	2 (2%)	0 (0%)	1 (12%)	0 (0%)	0 (0%)	0 (0%)	1 (2%)
Other	4 (3%)	0 (0%)	0 (0%)	0 (0%)	0 (0%)	0 (0%)	4 (8%)
Private	1 (1%)	0 (0%)	0 (0%)	1 (8%)	0 (0%)	0 (0%)	0 (0%)
Public	28 (24%)	0 (0%)	3 (38%)	2 (17%)	0 (0%)	13 (31%)	10 (21%)
*Trauma level*							
Absent	101 (86%)	4 (100%)	8 (100%)	6 (50%)	3 (100%)	35 (81%)	45 (94%)
Level 1	13 (11%)	0 (0%)	0 (0%)	4 (33%)	0 (0%)	8 (19%)	1 (2%)
Level 1;2	2 (2%)	0 (0%)	0 (0%)	1 (8%)	0 (0%)	0 (0%)	1 (2%)
Level 1;2;3	1 (1%)	0 (0%)	0 (0%)	1 (8%)	0 (0%)	0 (0%)	0 (0%)
Level 2	1 (1%)	0 (0%)	0 (0%)	0 (0%)	0 (0%)	0 (0%)	1 (2%)
*Multi-center*							
Multi-center	29 (26%)	0 (0%)	3 (38%)	7 (64%)	3 (100%)	33 (77%)	37 (84%)
Single-center	83 (74%)	3 (100%)	5 (62%)	4 (36%)	0 (0%)	10 (23%)	7 (16%)
Organizational Reporting Score (0-26), median (IQR)	10 (8, 13)	4 (3, 9)	11 (6, 13)	10 (8, 16)	9 (3, 10)	13 (9, 15)	9 (7, 11)

Comprehensive characterization of 118 included studies across six center types showing publication patterns, program establishment, patient volumes, study designs, hospital affiliations, and total domain scores. Orthoplastic center literature demonstrates the highest organizational feature reporting scores (median 13/26) with strong academic integration (67%) and trauma center affiliation (19%) despite smaller patient volumes compared to vascular centers

*Abbreviations*: N, Sample; IQR, Interquartile range

**Fig. 2:**
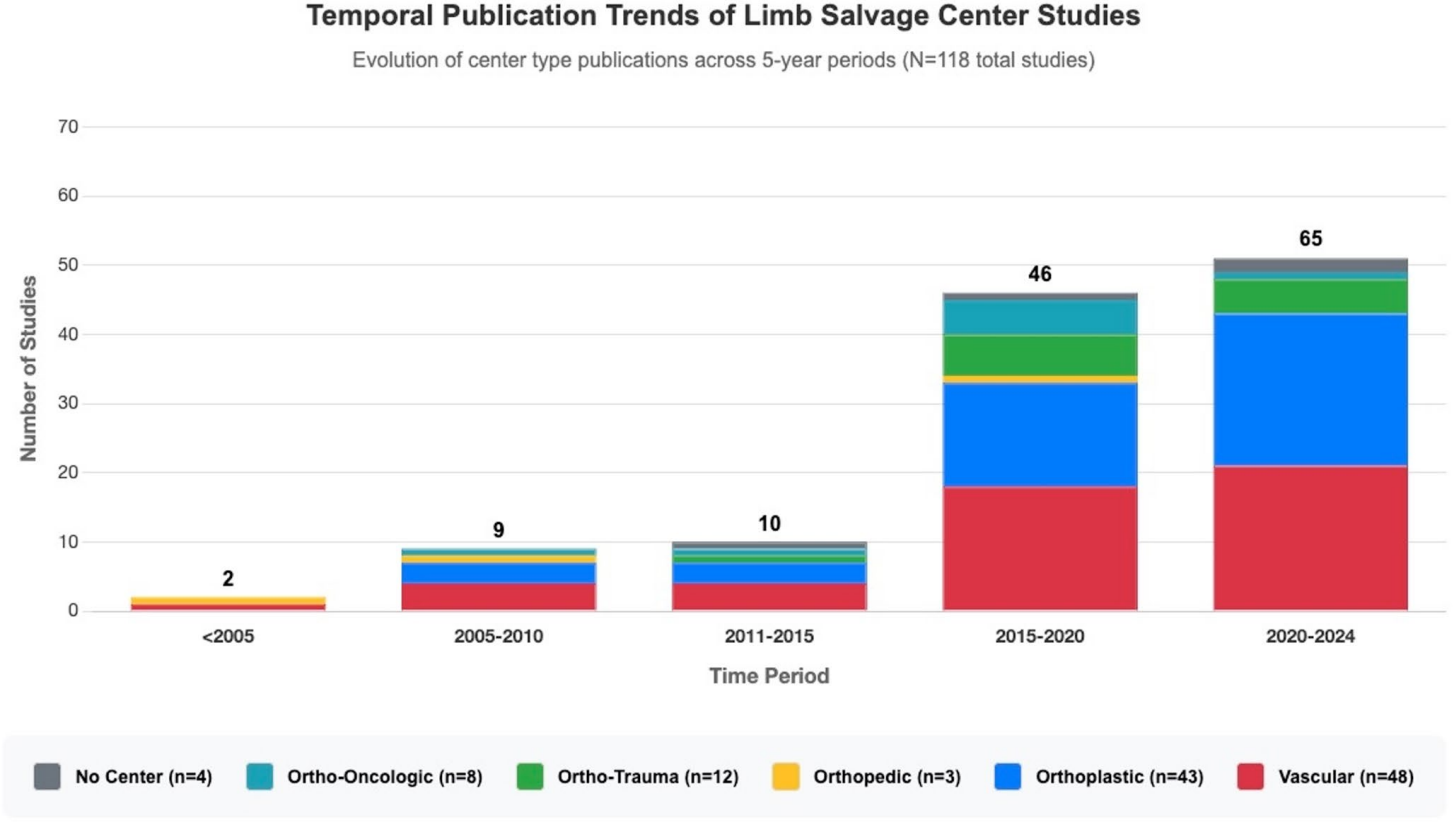
Temporal publication trends of limb salvage center studies by center type. Evolution of limb salvage center research from 1997–2024 across five-year periods, demonstrating substantial growth from 2 studies before 2005 to 65 studies in 2020–2024. Orthoplastic centers emerged from zero publications before 2005 to dominate recent literature (51% of 2020–2024 publications), while vascular center publications maintain consistent representation across all periods.

Publication analysis from 1997 to 2024 showed a 33-fold growth (Fig. 2), with the periods 2015–2020 (*n* = 46) and 2020–2024 (*n* = 65) accounting for 94% of all publications, and a median publication year of 2020 (IQR: 2015–2022). Orthoplastic center literature showed the largest growth, increasing from 0% of publications before 2005 to 34% of all publications in 2020–2024.

Studies came from 26 countries (Supplement 2), with the United Kingdom (UK) and United States of America (USA) accounting for 75% of all publications. The USA accounted for 49 studies (42%), predominantly describing vascular centers (*n* = 31, 63% of USA studies), while the UK contributed 39 studies (33%).

### Study characteristics

Published patient cohort sizes varied significantly across center types (*p* = 0.003). Vascular centers reported the largest patient populations by combining multiple limb preservation programs (median *n* = 406, IQR: 194–871), while orthoplastic centers reported smaller, more acute populations (median *n* = 101, IQR: 65–200) [22, 23].

The number of organizational features mentioned in publications varied significantly across center types (*p* < 0.001; Table 1). Publications by orthoplastic centers reported the most organizational features, with a total score of 50% [13/26 points], followed by vascular centers with 35% [9/26], ortho-oncologic 40% [11/26], and ortho-trauma centers 39% [10/26].

### Statistical differentiation of organizational features

**Fig. 3:**
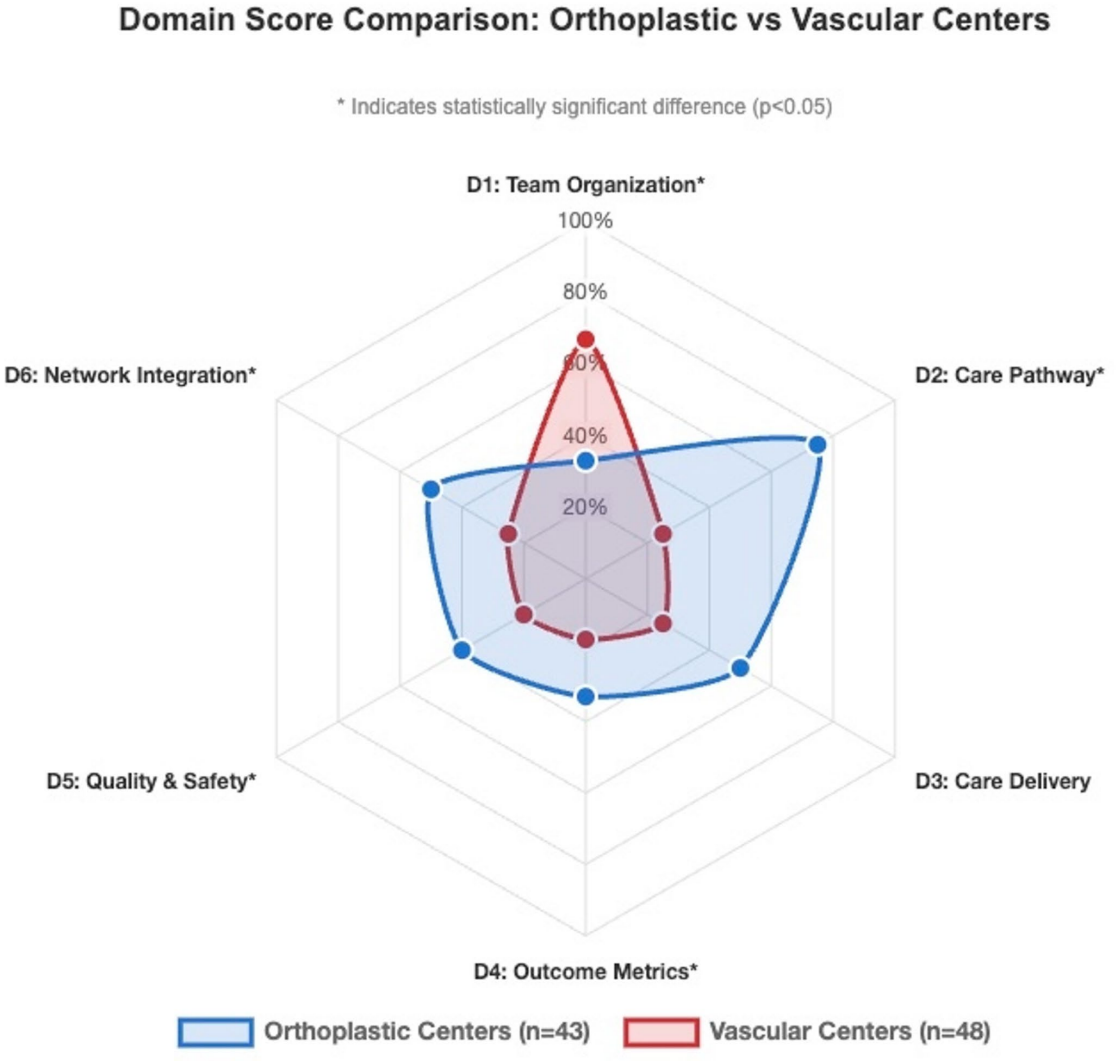
Comparison of the organizational reporting scores between orthoplastic and vascular limb salvage centers across six healthcare domains. Radar chart comparing reporting scores (%) across six structural care domains. Orthoplastic centers demonstrate statistically higher reporting scores in five domains, with the largest difference in care pathways (0.75 vs. 0.25, *p* < 0.001). Vascular center studies solely showed a higher reporting score in team organization documentation (0.67 vs. 0.33, *p* = 0.035), suggesting distinct limb center profiles.

Organizational feature comparison of the major center types (orthoplastic *n* = 43; vascular *n* = 48) demonstrated 44% higher median reporting scores by orthoplastic centers (13 vs. 9, *p* < 0.001) across five of six domains (Fig. 3). Studies describing vascular centers showed significantly higher reporting scores in the Team Organization domain (67% vs. 33%, *p* = 0.035).

A distinct comparison of all organizational features showed that 39% [10/26] were statistically different between orthoplastic and vascular center studies (Table 2).

**Table 2:** Statistical comparison of organizational features between orthoplastic and vascular centers

Domains and features	Orthoplastic (*n*=43)	Vascular (*n*=48)	Difference	*p*-value
*D1: Team and organization*				
Team composition documented	28 (65%)	45 (94%)	−29%	**<0.001**
Multidisciplinary meetings	15 (35%)	22 (46%)	−11%	0.29
Research team	11 (26%)	20 (42%)	−16%	0.11
*D2: Care pathways*				
Transfer protocols	28 (65%)	18 (38%)	+27%	**0.009**
Injury severity scoring	31 (72%)	33 (69%)	+3%	0.73
PRS Consult indication	38 (88%)	16 (33%)	+55%	**<0.001**
PRS Consult timing	32 (74%)	8 (17%)	+57%	**<0.001**
*D3: Care delivery*				
Emergency response protocol	18 (42%)	13 (27%)	+15%	0.14
Treatment algorithm	10 (23%)	13 (27%)	−4%	0.67
Standardized protocols	39 (91%)	43 (90%)	+1%	0.86
Socioeconomic disparity measures	4 (9%)	4 (8%)	+1%	0.87
*D4: Outcome metrics*				
Time to definitive treatment	19 (44%)	4 (8%)	+36%	**<0.001**
Time to coverage	19 (44%)	1 (2%)	+42%	**<0.001**
Psychological assessment	8 (19%)	4 (8%)	+11%	0.15
Functional assessment	21 (49%)	16 (33%)	+16%	0.13
Follow-up protocols	21 (49%)	16 (33%)	+16%	0.13
Long-term complication monitoring	26 (60%)	27 (56%)	+4%	0.68
*D5: Quality and safety*				
Case audit frequency	4 (9%)	2 (4%)	+5%	0.32
Quality metrics	31 (72%)	43 (90%)	−18%	**0.033**
Morbidity/mortality reviews	3 (7%)	2 (4%)	+3%	0.56
Adverse event tracking	29 (67%)	12 (25%)	+42%	**<0.001**
External certification	2 (5%)	2 (4%)	+1%	0.91
*D6: Network integration*				
Centralized care	35 (81%)	26 (54%)	+27%	**0.006**
Trauma network participation	16 (37%)	2 (4%)	+33%	**<0.001**
National limb salvage registry	4 (9%)	5 (10%)	−1%	0.86
National policy compliance	22 (51%)	17 (35%)	+16%	0.13
*Overall organizational score*				
*Total domain score*,* median (IQR)*	*13 (9*,* 15)*	*9 (7*,* 11)*	*+4 points*	***<0.001***

Comprehensive analysis of 26 structural elements across six domains comparing orthoplastic (*n* = 43) and vascular (*n* = 48) centers. Ten elements show statistically significant differences (*p* < 0.05), with orthoplastic centers demonstrating superiority in care pathways (PRS integration), timing metrics, and network integration, while vascular centers excel in team documentation and quality metrics reporting

Statistical Tests: Pearson’s chi-squared test for categorical variables; Wilcoxon rank-sum test for continuous variables

Significant findings (*p* < 0.05) highlighted: Team composition, transfer protocols, PRS consult indication, PRS consultation timing, timing metrics, quality metrics, adverse event tracking, centralized care, and trauma network participation

Literature describing orthoplastic centers demonstrated higher organizational feature scores in Care Pathways: plastic and reconstructive surgery (PRS) consultation criteria (88% vs. 33%, *p* < 0.001), PRS consultation timing (74% vs. 17%, *p* < 0.001), and transfer protocols (65% vs. 38%, *p* = 0.009). Outcome Metrics showed higher scores for timing documentation: time to definitive treatment (44% vs. 8%, *p* < 0.001) and time to soft tissue coverage (44% vs. 2%, *p* < 0.001). Network Integration mentions were more common in orthoplastic centers through increased trauma network participation (37% vs. 4%, *p* < 0.001) and centralized care models (81% vs. 54%, *p* = 0.006). Quality and Safety measures showed higher adverse event tracking systems (67% vs. 25%, *p* < 0.001).

Conversely, vascular center literature more frequently reported Team Organization: team composition documentation (94% vs. 65%, *p* < 0.001) and Quality and Safety: quality metrics tracking (90% vs. 72%, *p* = 0.033), reflecting established framework documentation.

### Team and service architecture

**Fig. 4:**
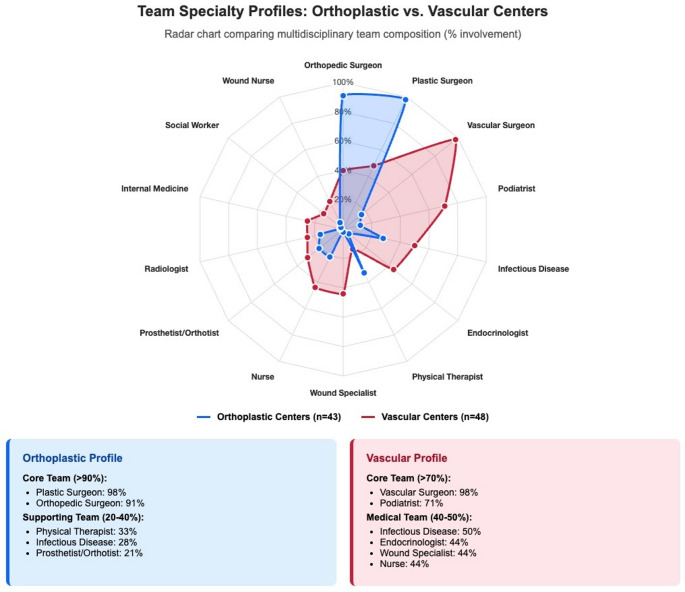
Multidisciplinary team composition profiles comparing orthoplastic and vascular centers. Radar chart illustrating distinct team architecture patterns, implying different care philosophies. Orthoplastic centers feature surgical reconstruction teams (98% plastic surgeon + 91% orthopaedic surgeon) with rehabilitation focus, while vascular centers emphasize medical management teams (98% vascular surgeon + 71% podiatrist) with comorbidity optimization specialists.

Team architecture reporting score comparison between studies describing orthoplastic centers vs. vascular centers revealed distinct multidisciplinary care models (Fig. 4). of orthoplastic centers (*n* = 38/43) featured surgical reconstruction-focused teams including plastic surgeons (98%) and orthopaedic surgeons (91%), in a combined (orthoplastic) surgical approach. Supporting members emphasized rehabilitation care models, including physical therapists (33%) and prosthetists/orthotists (21%).

Studies by vascular centers mainly describe medical management teams, with vascular surgeons (98%) and podiatrists (71%) forming the core of these centers. Medical specialists were prevalently reported, including infectious disease physicians (50%), endocrinologists (44%), and wound care specialists (44%). Physical therapist mentions were substantially lower (15%) than in orthoplastic center studies.

### Universal orthoplastic center characteristics

Analysis of orthoplastic center publications identified six features reported in > 70% of studies: standardized care protocols (91%), PRS consultation criteria (88%), PRS consultation timing (74%), centralized care delivery (81%), quality metrics tracking (72%), and injury severity scoring (72%).

### Individual study analysis

Individual study analysis revealed heterogeneous reporting rates of organizational features within center types *(Supplement 1).* Orthoplastic centers demonstrated reporting scores ranging from 19 to 65%, while vascular centers ranged from 4 to 54%. Seven orthoplastic centers achieved total scores exceeding 60%, compared to one vascular center exceeding 50%. Overall, the highest scoring studies described formalized limb salvage care pathways, such as the orthoplastic approach in the British Orthopaedic Association Standards for Trauma (BOAST) national guidelines [24, 25].

### Risk of bias assessment

Risk of bias assessment using the MINORS tool for non-randomized studies, the Joanna Briggs Institute (JBI) critical appraisal tools for cross-sectional studies, cohort studies, case series, and text and opinion articles, the ROBIS tool for systematic reviews, and the SANRA scale for narrative reviews demonstrated moderate methodological quality across studies(Supplement 3). While most studies demonstrated low to moderate risk in outcome measurement and reporting, common limitations included retrospective designs, inconsistent outcome protocols, and potential selection bias in single-center studies.

## Discussion

**Fig. 5:**
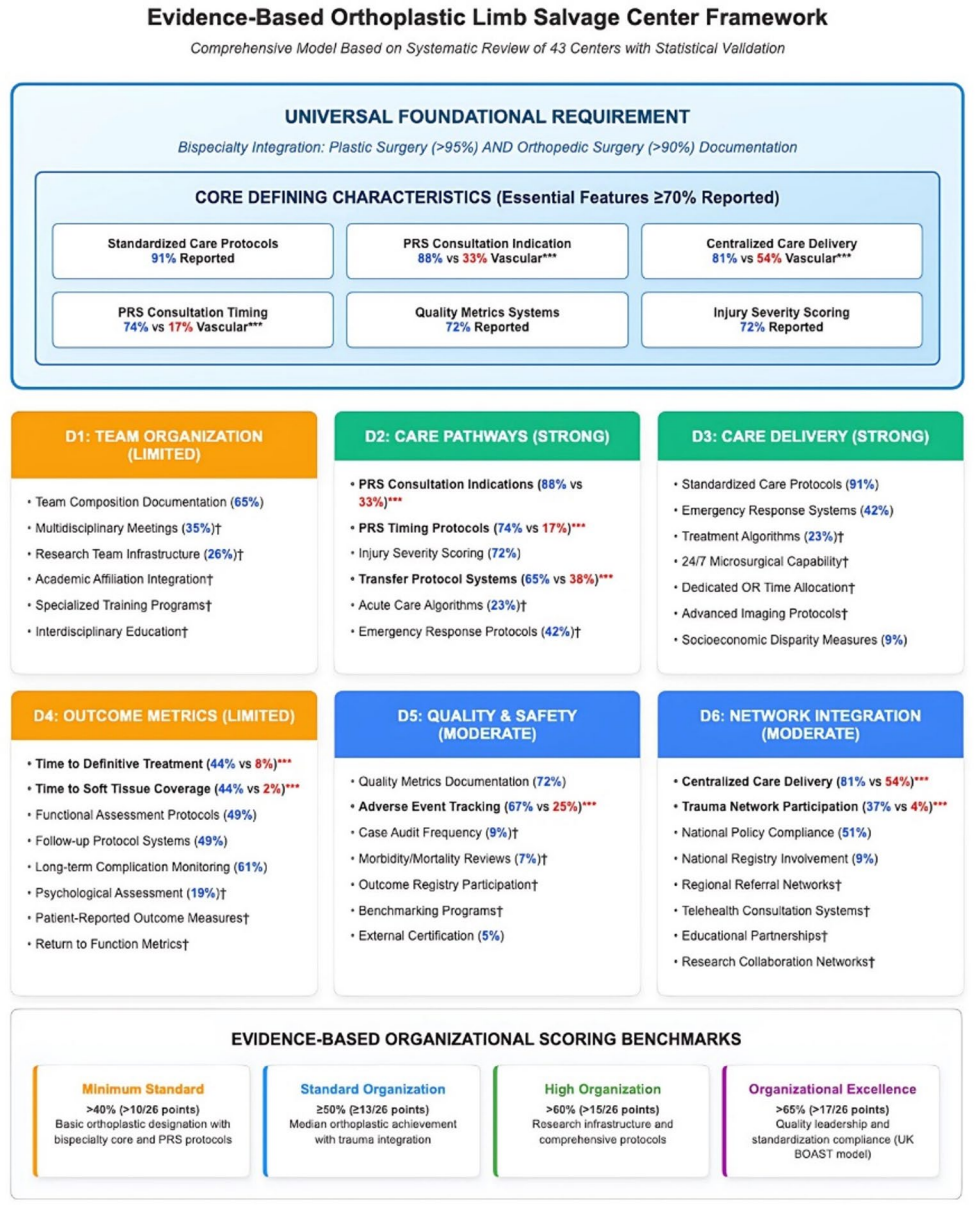
Evidence-Based Orthoplastic Limb Salvage Center Framework. Organizational feature prevalence across 43 orthoplastic centers, with feature reporting score benchmarks across six domains. Six core defining organizational features demonstrated > 70% reporting prevalence: standardized care protocols (91%), PRS consultation criteria (88%), PRS consultation timing (74%), centralized care delivery (81%), quality metrics tracking (72%), and injury severity scoring (72%). Statistical analysis reveals significantly higher reporting scores for orthoplastic centers in 10 organizational features compared to vascular centers (*p* < 0.05), supporting the need for distinct standardization frameworks. *** Statistically Significant: Features with *p* < 0.05 compared to vascular centers. † Enhanced Elements: Development opportunities with substantial standardization gaps. Domain Strength Coding: *Strong* (≥ 1 feature > 75% or > 90%), *Moderate* (1 feature > 70%), *Limited* (no features > 70%). Statistical Values: *Blue* represents orthoplastic rates, *red* represents vascular comparison data.

This systematic review of 118 studies demonstrates that orthoplastic centers differ significantly from vascular centers in 10 of 26 organizational features across 5 of 6 structural domains, suggesting distinct care models that may benefit from standardized frameworks. Based on the most prevalently reported organizational features in orthoplastic center literature, our framework suggests universal benchmarks and priority areas that may enhance limb salvage care quality (Fig. 5).

Studies describing orthoplastic centers revealed a care model built around bi-specialty integration and acute reconstruction principles encompassing plastic surgeons (98%) and orthopaedic surgeons (91%). These centers included physical therapists (33%) and prosthetists/orthotists (21%), reflecting a focus on complex tissue reconstruction and rehabilitation [26].

The orthoplastic approach, defined and popularized by Levin in 1993 as combined reconstructive care [27], has evolved into a specialized care model with unique organizational characteristics. The lack of standardized benchmarks may be related to the inherently challenging heterogeneous nature of reconstructive approaches [28]. Six features demonstrated reporting prevalence > 70% [29], suggesting core organizational features: standardized care protocols (91%), PRS consultation criteria (88%), PRS consultation timing (74%), centralized care delivery (81%), quality metrics tracking (72%), and injury severity scoring (72%).

Organizational strengths of orthoplastic centers reflect their focus on acute, time-sensitive interventions. Significantly higher reporting scores for PRS consultation criteria and timing frameworks align with evidence that early combined reconstruction significantly improves outcomes for severe open fractures [17, 30, 31]. Transfer protocols were more frequently reported, supporting findings that early transfer to specialized trauma centers may improve surgical outcomes, as reported before in the Lower Extremity Guidelines for Salvage (L.E.G.S) [15, 23]. Trauma network participation and centralized care models were more prevalent, consistent with evidence supporting specialized orthoplastic approaches for complex reconstruction [9, 32, 33].

However, orthoplastic center literature revealed potential organizational limitations. Team composition documentation was significantly less frequently reported than in vascular center literature (*p* < 0.001). Research infrastructure appeared less developed compared to vascular centers. Patient-reported outcome measure collection, essential for quality improvement, faces implementation challenges in high-volume multidisciplinary centers [34]. Orthoplastic center literature also significantly reported quality metrics less frequently. Unlike vascular limb salvage centers, which have benefited from systematic quality frameworks, orthoplastic centers have developed without universal standardization despite documented clinical advantages [35–37]. The absence of orthoplastic standardization guidelines, in contrast with the well-documented frameworks in vascular center literature, suggests a need for more comprehensive organizational limb salvage center frameworks.

Literature describing vascular centers revealed a more established care model emphasizing chronic disease management and systematic documentation practices. Significant differences in organizational features showed that vascular studies more frequently documented team organization and quality systems, reflecting established frameworks that have evolved through decades of peripheral arterial disease and diabetic foot management [10, 38].

Team composition was significantly more frequently reported, indicating well-defined multidisciplinary structures that support comprehensive limb salvage alliances [39, 40]. Studies describing vascular centers reported teams with vascular surgeons (98%) and podiatrists (71%), along with other medical specialties, including infectious diseases (50%), endocrinology (44%), and wound care (44%) [8].

Organizational strengths of vascular centers stem from their foundation in systematic chronic disease management and quality improvement initiatives [10]. Quality Metrics reporting was significantly higher than in orthoplastic center literature, reflecting established continuous improvement processes enabled by institutionalized quality systems [10]. These centers benefit from established clinical guidelines that define clear criteria for interventions [35, 41]. The economic value of specialized services within limb salvage alliances has been well-documented, supporting evidence for systematic organization [39]. In our findings, research infrastructure is more developed in vascular centers, with systematic approaches to program development and implementation [40]. This foundation has enabled evidence-based protocol development and outcome evaluation, contributing to established intervention guidelines and care pathways for vascular limb salvage.

However, vascular studies revealed potential limitations in acute care coordination. Lower reporting scores for timing metrics and transfer protocols may suggest lower responsiveness to acute limb-threatening scenarios requiring immediate intervention. The often less acute nature of vascular interventions, while allowing for systematic planning, may not optimize care for patients requiring emergent limb salvage procedures.

Vascular center studies also demonstrated substantial heterogeneity in organizational feature reporting scores (4 to 54%), warranting consideration for the discrimination of preventive limb preservation and surgical vasculoplastic centers [42–44].

Our findings show that orthoplastic and vascular centers have evolved distinct organizational approaches that directly impact patient outcomes [13, 45–47]. Orthoplastic centers demonstrated significantly higher reporting in domains important for acute trauma care: Care Pathways (timing protocols), Outcome Metrics (functional assessments), and Network Integration (trauma system participation). This organizational focus on coordinated intervention aligns with clinical evidence showing that direct admission to specialized trauma centers and adherence to timing protocols significantly improve outcomes in severe open fractures, optimized for acute interventions requiring rapid multidisciplinary coordination [23, 48, 49].

However, these organizational advantages are inconsistently implemented. Orthoplastic center studies demonstrated substantial variability in organizational feature reporting scores (19 to 65% range), with only seven orthoplastic centers achieving scores of > 60%. The highest-performing centers follow established national guidelines, while others lack systematic frameworks [30, 50–52]. However, even in centers with long-established national orthoplastic care protocols, adherence has proven to be challenging due to organizational limitations [19, 53–55]. This suggests that formalized guideline designation alone does not ensure standardized care delivery, highlighting the need for organizational structure to fully realize the timely needs of the orthoplastic approach.

The temporal evolution of orthoplastic care reveals increasing reporting in the literature over time. From zero publications < 2005, orthoplastic centers now represent 51% of recent limb salvage center literature (2020–2024), indicating greater interest in the literature. This growth reflects recognition of orthoplastic principles in limb reconstruction [1]. Current evidence strongly supports the clinical benefits of coordinated orthoplastic approaches. Interdisciplinary networks demonstrate improved outcomes for complex patient populations, with specialized centers providing coordinated care and enhanced access [26].

Geographic patterns provide insights into healthcare system influences on standardization. The UK’s leadership in orthoplastic research (53% of global literature) corresponds with the implementation of national BOAST guidelines in 2008, which have provided systematic orthoplastic frameworks for open fracture management [19, 48]. Studies demonstrate that the transition from guidelines to standards of care requires systematic implementation and monitoring, with evidence that structured approaches improve care consistency [48]. However, multiple audit studies of orthoplastic centers demonstrate variable compliance with national guidelines, even among specialized institutions [19, 53–56]. For example, a recent international study of 62 centers highlights significant disparities in limb salvage care globally, which can benefit from global quality improvement collaboration [55]. Subsequent qualitative analysis reported orthoplastic guideline implementation barriers that include individualistic decision-making and reliance on interpersonal relationships rather than systematic protocols, because non-compliance currently has no consequences [54]. These recent developments suggest a need for collaborative quality initiatives, requiring universal orthoplastic center standardization, to potentially benefit from cost-effective optimization of surgical outcomes seen in other disciplines [57–60].

Priority domains for standardization include: (1) comprehensive outcome measurement systems, (2) organizational team structure, (3) quality and safety systems, and (4) centralized network integration (Supplement 4). Respecting healthcare system and center variability, our proposed organizational features may allow orthoplastic center development tailored to their timely needs, sustainably optimizing limb salvage outcomes [19, 61].

Future efforts may benefit from academic center experiences and practices to develop standardized benchmarks for quality improvement initiatives. Implementation studies evaluating framework effectiveness could provide evidence for broader adoption [54]. These studies should incorporate validated measures of functional outcomes, quality of life, and long-term limb salvage outcomes. Alignment with commonly used metrics may enable more rigorous outcome comparisons [34, 62]. Consensus studies of orthoplastic center practices could facilitate evidence-based framework development that prioritizes patient outcomes while remaining practical for diverse clinical settings.

These results should be interpreted within the context of the study design. Most importantly, this analysis reflects reporting patterns in published literature rather than actual organizational characteristics of centers. However, academic centers comprised 62% of our sample and comprehensively described organizational descriptions, making under-reporting less likely. Geographic clustering (75% USA/UK studies) may limit generalizability, and publication bias may favor centers with more developed structures. Our binary coding system may not capture implementation nuances, and center classification relied on author-reported designations. This study evaluated organizational descriptions rather than patient outcomes, warranting future assessment of how structural differences impact limb salvage success rates.

An additional conceptual limitation warrants explicit acknowledgment. ‘Limb salvage’ is an umbrella term that spans clinically distinct entities – acute traumatic injury, chronic limb-threatening ischemia, diabetic foot complications, and oncologic reconstruction – each of which requires a different care model. Although our center-type stratification broadly reflects these differing dominant indications, residual within-category heterogeneity persists (e.g., orthoplastic centers may manage both open fractures and oncologic reconstruction). A single aggregated, score-based definition applicable across all limb-threatening pathologies may therefore not be feasible based on the available literature. The organizational framework we propose is intended primarily for orthoplastic limb salvage centers, which predominantly serve acute trauma and complex reconstructive populations; indication-specific frameworks may ultimately be needed for the full spectrum of limb salvage care.

## Conclusion

This systematic review of limb salvage center literature demonstrates that orthoplastic centers show significantly different organizational features from vascular centers across structural care domains, indicating that distinct care models may require unique standardization frameworks. In contrast with vascular center literature, these remain absent for orthoplastic limb salvage centers. The substantial heterogeneity between orthoplastic center studies suggests opportunities for standardization that may optimize care delivery. Besides orthoplastic surgery, six characterizing organizational features with > 70% prevalence could serve as orthoplastic center benchmarks for quality improvement initiatives, respecting limb salvage center variability. Our findings suggest a framework with standardization priorities for orthoplastic center development that may improve limb salvage outcomes.

## Supplementary Information

Below is the link to the electronic supplementary material.

Supplement 0. Search syntax table. Search syntax across databases for studies on limb salvage/reconstructive programs and orthoplastic/oncoplastic care models. Syntax adapted to database-specific conventions (e.g., MeSH/Emtree, proximity operators). All searches executed on 12/2024 by Harvard Countway Library.

Supplement 1. Complete study characteristics and domain score profiles for all included limb salvage center studies. Comprehensive individual study data for all 118 included studies showing detailed breakdown by center type, geographic distribution, methodological characteristics, and complete domain scoring (D1-D6). Data demonstrates wide performance variation within center types (4%-69 % total scores) and enables granular analysis of center-specific patterns across organizational domains. Domain score transparency supports reproducibility and enables identification of high-performing centers for benchmarking purposes.

Supplement 2. Geographic distribution of limb salvage center studies by country and center type. Global distribution of 118 studies across 26 countries showing clear geographic specialization patterns. USA dominates vascular research (65% of global vascular literature; 31/48 studies) while UK leads orthoplastic research (53% of global orthoplastic literature; 23/43 studies), indicating healthcare system-influenced practice development with emerging contributions from 22 additional countries.

Supplement 3. Risk of bias assessment. Risk of bias assessment using the appropriate validated tools across studies. While most studies demonstrated low to moderate risk in outcome measurement and reporting, common limitations included retrospective designs, inconsistent outcome protocols, and potential selection bias in single-center studies.

Supplement 4. Orthoplastic center standardization priorities. Standardization priorities for orthoplastic center organizational features ranked by implementation gap. CRITICAL priorities (red, >75% gap) require immediate consensus development; HIGH priorities (orange, 50–75% gap) need structured standardization; MODERATE priorities (blue, 25–50% gap) require refinement of existing practices; ADEQUATE priorities (green, <25% gap) need validation and specification only. Gap percentages represent the difference between current reporting rates and optimal 100% implementation.

## Data Availability

The data used are available from the corresponding author upon reasonable request.
