# Supplementary material for: Defining orthoplastic limb salvage centers: a systematic review
Source: Arch Orthop Trauma Surg. 2026 May 2;146(1):171. doi: 10.1007/s00402-026-06325-0 (PMC13135554; doi:10.1007/s00402-026-06325-0)
Supplement: Supplementary file 1 — Supplement 0. Search syntax table. Search syntax across databases for studies on limb salvage/reconstructive programs and orthoplastic/oncoplastic care models. Syntax adapted to database-specific conventions (e.g., MeSH/Emtree, proximity operators). All searches executed on 12/2024 by Harvard Countway Library. [file 402_2026_6325_MOESM1_ESM.docx]

| **Component** | **MEDLINE (PubMed)** | **Embase** | **Web of Science** | **Cochrane** |
| --- | --- | --- | --- | --- |
| **Structure Filter** | exp Limb Salvage/ AND (Hospital Units/ OR exp Hospitals/ OR exp Trauma Centers/) | 'limb salvage'/exp AND ('hospital subdivisions and components'/exp OR 'hospital'/exp OR 'emergency health service'/exp) | *N/A* | *N/A* |
| **Limb Intervention Terms** | ((limb? OR extremit*) adj3 (salvag* OR reconstruct* OR preserv* OR restor*)).ti,ab,kw,kf | (('limb$' OR 'extremit*') NEAR/3 ('salvag*' OR 'reconstruct*' OR 'preserv*' OR 'restor*')):ti,ab,kw | TI=(("limb?" OR "extremit*") NEAR/3 ("salvag*" OR "reconstruct*" OR "preserv*" OR "restor*")) OR AB=(...) OR AK=(...) | ((limb? OR extremit*) NEXT/3 (salvag* OR reconstruct* OR preserv* OR restor*)) |
| **Program/Setting Terms** | ((salvag* OR reconstruct* OR preserv* OR restor*) adj3 (program* OR center? OR centre? OR hospital? OR institute? OR unit? OR department? OR service? OR clinic?)).ti,ab,kw,kf | (('salvag*' OR 'reconstruct*' OR 'preserv*' OR 'restor*') NEAR/3 ('program*' OR 'center$' OR 'centre$' OR 'hospital$' OR 'institute$' OR 'unit$' OR 'department$' OR 'service$' OR 'clinic$')):ti,ab,kw | TI=(("salvag*" OR "reconstruct*" OR "preserv*" OR "restor*") NEAR/3 ("program*" OR "center?" OR "centre?" OR "hospital?" OR "institute?" OR "unit?" OR "department?" OR "service?" OR "clinic?")) OR AB=(...) OR AK=(...) | ((salvag* OR reconstruct* OR preserv* OR restor*) NEXT/3 (program* OR center? OR centre? OR hospital? OR institute? OR unit? OR department? OR service? OR clinic?)) |
| **Orthoplastic Terms** | ((orthoplastic? OR ortho plastic? OR orthop?edic plastic? OR oncoplastic? OR onco plastic?) adj3 (program* OR center? OR centre? OR hospital? OR institute? OR unit? OR department? OR service? OR clinic?)).ti,ab,kf,kw | (('orthoplastic$' OR 'ortho plastic$' OR 'orthop$edic plastic$' OR 'oncoplastic$' OR 'onco plastic$') NEAR/3 ('program*' OR 'center$' OR 'centre$' OR 'hospital$' OR 'institute$' OR 'unit$' OR 'department$' OR 'service$' OR 'clinic$')):ti,ab,kw | TI=(("orthoplastic*" OR "ortho plastic*" OR "orthopedic plastic*" OR "orthopaedic plastic*" OR "oncoplastic*" OR "onco plastic*") NEAR/3 ("program*" OR "center*" OR "centre*" OR "hospital*" OR "institute*" OR "unit*" OR "department*" OR "service*" OR "clinic*")) OR AB=(...) OR AK=(...) | ((orthoplastic? OR ortho plastic? OR orthop?edic plastic? OR oncoplastic? OR onco plastic?) NEXT/3 (program* OR center? OR centre? OR hospital? OR institute? OR unit? OR department? OR service? OR clinic?)) |
| **Final Syntax** | 1 OR (2 AND 3) OR 4 | 1 OR (2 AND 3) OR 4 | (1 AND 2) OR 3 | (1 AND 2) OR 3 |

***Supplement 0.* Search Syntax Table.***Search syntax across databases for studies on limb salvage/reconstructive programs and orthoplastic/oncoplastic care models. Syntax adapted to database-specific conventions (e.g., MeSH/Emtree, proximity operators). All searches executed on 12/2024 by Harvard Countway Library.*
