# Supplementary material for: Defining orthoplastic limb salvage centers: a systematic review
Source: Arch Orthop Trauma Surg. 2026 May 2;146(1):171. doi: 10.1007/s00402-026-06325-0 (PMC13135554; doi:10.1007/s00402-026-06325-0)
Supplement: Supplementary file 2 — Supplement 1. Complete study characteristics and domain score profiles for all included limb salvage center studies. Comprehensive individual study data for all 118 included studies showing detailed breakdown by center type, geographic distribution, methodological characteristics, and complete domain scoring (D1-D6). Data demonstrates wide performance variation within center types (4%-69 % total scores) and enables granular analysis of center-specific patterns across organizational domains. Domain score transparency supports reproducibility and enables identification of high-performing centers for benchmarking purposes. [file 402_2026_6325_MOESM2_ESM.docx]

| **Study ID** | **Study** | **Journal** | **Country** | **Study Design** | | **N** | **Program /Center Establishment Year** | **Type of Center** | **Hospital Type** | **Trauma Level** | **Multi-Center** | **Organizational Feature Reporting Score** | **Reporting Score (%)** |
| --- | --- | --- | --- | --- | --- | --- | --- | --- | --- | --- | --- | --- | --- |
| 1 | Berner, 2024 | Injury-International Journal of the Care of the Injured | Global | Other | |  |  | No center | Absent | Absent | Multi-center | D1:0, D2:1, D3:0, D4:0, D5:0, D6:0 (Total: 1/26) | 3.85% |
| 2 | Mcculloch, 2015 | Annals of Surgery | UK | Prospective cohort | |  |  | No center | Academic | Absent | Multi-center | D1:1, D2:1, D3:1, D4:0, D5:1, D6:0 (Total: 4/26) | 15.38% |
| 3 | Yeung, 2021 | Spinal Cord | USA; canada | Review | |  |  | No center | Academic | Absent | Multi-center | D1:2, D2:1, D3:0, D4:0, D5:0, D6:1 (Total: 4/26) | 15.38% |
| 4 | Langer, 2014 | The scientific world journal | India | Review | |  |  | No center | Military | Absent |  | D1:1, D2:4, D3:2, D4:4, D5:1, D6:1 (Total: 13/26) | 50.00% |
| 5 | Abarca, 2018 | Journal of Surgical Oncology | United states | Retrospective cohort | | 4874 |  | Ortho-oncologic | Absent | Absent | Multi-center | D1:1, D2:0, D3:1, D4:1, D5:1, D6:2 (Total: 6/26) | 23.08% |
| 6 | Morris, 2020 | JAAOS: Global Research and Reviews | Haiti; Dominican republic | Case series | |  |  | Ortho-oncologic | Public | Absent | Multi-center | D1:1, D2:1, D3:1, D4:2, D5:1, D6:0 (Total: 6/26) | 23.08% |
| 7 | Malik, 2020 | Clinical Orthopaedics & Related Research | USA | Retrospective cohort | | 14039 |  | Ortho-oncologic | Academic | Absent | Multi-center | D1:1, D2:1, D3:0, D4:1, D5:1, D6:2 (Total: 6/26) | 23.08% |
| 8 | Zeller, 2019 | Frontiers in Oncology | Germany | Retrospective cohort | | 48 |  | Ortho-oncologic | Academic | Absent | Single-center | D1:2, D2:2, D3:1, D4:2, D5:2, D6:1 (Total: 10/26) | 38.46% |
| 9 | Thomas, 2022 | Cancers | Germany | Prospective cohort | | 621 |  | Ortho-oncologic | Multiple | Absent | Multi-center | D1:2, D2:2, D3:1, D4:1, D5:2, D6:3 (Total: 11/26) | 42.31% |
| 10 | Leckenby, 2017 | Annals of Plastic Surgery | UK | Retrospective cohort | | 298 |  | Ortho-oncologic | Public | Absent | Single-center | D1:2, D2:1, D3:2, D4:5, D5:2, D6:1 (Total: 13/26) | 50.00% |
| 11 | Haidar, 2008 | Pediatric Blood & Cancer | Lebanon | Retrospective cohort | | 30 | 2002 | Ortho-oncologic | Academic | Absent | Multi-center | D1:3, D2:2, D3:2, D4:3, D5:2, D6:1 (Total: 13/26) | 50.00% |
| 12 | Gaston, 2014 | Bone and Joint Journal | UK | Retrospective cohort | | 95 |  | Ortho-oncologic | Public | Absent | Single-center | D1:2, D2:3, D3:1, D4:4, D5:2, D6:2 (Total: 14/26) | 53.85% |
| 13 | Noorlander-Borgdorff, 2024 | Injury | Netherlands | Retrospective cohort | | 1079 | 2017 | Ortho-trauma | Academic | Level 1;2;3 | Multi-center | D1:1, D2:1, D3:3, D4:1, D5:1, D6:0 (Total: 7/26) | 26.92% |
| 14 | Franz, 2012 | Annals of Vascular Surgery | USA | Retrospective cohort | | 135 |  | Ortho-trauma | Academic | Level 1 | Single-center | D1:1, D2:2, D3:3, D4:1, D5:0, D6:0 (Total: 7/26) | 26.92% |
| 15 | Mullis, 2017 | JBJS Reviews | North America | Review | |  |  | Ortho-trauma | Academic | Absent |  | D1:0, D2:3, D3:1, D4:0, D5:1, D6:2 (Total: 7/26) | 26.92% |
| 16 | Izawa, 2024 | Journal of Orthopaedic Science | Japan | Case series | | 35 | 2019 | Ortho-trauma | Private | Absent | Single-center | D1:1, D2:1, D3:2, D4:1, D5:2, D6:1 (Total: 8/26) | 30.77% |
| 17 | Rozbruch, 2015 | Clinical Orthopaedics & Related Research | USA | Retrospective cohort | | 672 | 2005 | Ortho-trauma | Academic | Absent | Single-center | D1:3, D2:1, D3:1, D4:0, D5:3, D6:1 (Total: 9/26) | 34.62% |
| 18 | Pari, 2021 | Acta Bio-Medica de l Ateneo Parmense | Italy | Retrospective cohort | | 74 |  | Ortho-trauma | Public | Absent | Single-center | D1:0, D2:3, D3:2, D4:3, D5:2, D6:0 (Total: 10/26) | 38.46% |
| 19 | Bunn, 2021 | Journal of the American College of Surgeons | USA | Retrospective cohort | | 712 |  | Ortho-trauma | Academic | Level 1;2 | Multi-center | D1:1, D2:2, D3:1, D4:0, D5:2, D6:4 (Total: 10/26) | 38.46% |
| 20 | Hohmann, 2017 | Injury | South Africa, uae, USA, Australia | Case series | | 32 |  | Ortho-trauma | Academic | Absent | Single-center | D1:1, D2:2, D3:1, D4:4, D5:1, D6:2 (Total: 11/26) | 42.31% |
| 21 | Khadim, 2019 | Injury | UK | Retrospective cohort | | 132 |  | Ortho-trauma | Public | Absent | Multi-center | D1:1, D2:3, D3:2, D4:5, D5:2, D6:3 (Total: 16/26) | 61.54% |
| 22 | Kim, 2022 | BMC Emergency Medicine | Korea | Retrospective cohort | | 273 | 2016 | Ortho-trauma | Academic | Level 1 | Single-center | D1:2, D2:4, D3:2, D4:2, D5:3, D6:3 (Total: 16/26) | 61.54% |
| 23 | Messner, 2020 | Injury | UK | Retrospective cohort | | 32 | 2013 | Ortho-trauma | Academic | Level 1 | Single-center | D1:1, D2:4, D3:2, D4:6, D5:1, D6:3 (Total: 17/26) | 65.38% |
| 24 | Baus, 2020 | Annales de Chirurgie Plastique et Esthetique | France | Retrospective cohort | | 223 |  | Ortho-trauma | Military | Level 1 | Multi-center | D1:2, D2:4, D3:3, D4:4, D5:2, D6:3 (Total: 18/26) | 69.23% |
| 25 | Bond, 1999 | Child: Care, Health & Development | UK | Cross-sectional | | 53 |  | Orthopedic | Academic | Absent | Single-center | D1:2, D2:0, D3:0, D4:1, D5:0, D6:0 (Total: 3/26) | 11.54% |
| 26 | Pinzur, 2009 | Orthopedics | USA | Retrospective cohort | | 86 | 2007 | Orthopedic | Academic | Absent | Single-center | D1:2, D2:1, D3:2, D4:0, D5:3, D6:1 (Total: 9/26) | 34.62% |
| 27 | Hruby, 2017 | Journal of Neurosurgery | Austria | Retrospective cohort | | 34 |  | Orthopedic | Academic | Absent | Single-center | D1:1, D2:2, D3:2, D4:4, D5:1, D6:0 (Total: 10/26) | 38.46% |
| 28 | Fan, 2021 | Plastic and Reconstructive Surgery - Global Open | USA | Retrospective cohort | | 25415 |  | Orthoplastic | Public | Absent | Multi-center | D1:1, D2:2, D3:1, D4:1, D5:0, D6:0 (Total: 5/26) | 19.23% |
| 29 | Abu el Hawa, 2022 | Advances in Wound Care | USA | Retrospective cohort | | 64 |  | Orthoplastic | Academic | Absent | Single-center | D1:1, D2:2, D3:1, D4:0, D5:1, D6:0 (Total: 5/26) | 19.23% |
| 30 | Radotra, 2022 | Injury-International Journal of the Care of the Injured | UK | Cross-sectional | |  | 2012 | Orthoplastic | Academic | Level 1 | Multi-center | D1:0, D2:2, D3:1, D4:0, D5:1, D6:3 (Total: 7/26) | 26.92% |
| 31 | Hadfield, 2022 | Bone & Joint Open | UK | Prospective cohort | | 1175 |  | Orthoplastic | Academic | Absent | Multi-center | D1:2, D2:3, D3:0, D4:1, D5:0, D6:1 (Total: 7/26) | 26.92% |
| 32 | Strong, 2020 | Injury | UK | Retrospective cohort | | 24 |  | Orthoplastic | Academic | Level 1 | Single-center | D1:1, D2:1, D3:0, D4:1, D5:2, D6:3 (Total: 8/26) | 30.77% |
| 33 | Sharif-Askary, 2021 | Plastic & Reconstructive Surgery | USA | Retrospective cohort | | 200 |  | Orthoplastic | Academic | Absent | Single-center | D1:2, D2:2, D3:1, D4:1, D5:1, D6:1 (Total: 8/26) | 30.77% |
| 34 | Bridgeman, 2022 | Missouri Medicine | USA | Review | |  |  | Orthoplastic | Academic | Absent | Single-center | D1:1, D2:3, D3:1, D4:0, D5:0, D6:3 (Total: 8/26) | 30.77% |
| 35 | Sayyed, 2024 | Journal of the American Podiatric Medical Association | USA | Other | | 420 |  | Orthoplastic | Academic | Absent | Single-center | D1:2, D2:2, D3:1, D4:1, D5:1, D6:1 (Total: 8/26) | 30.77% |
| 36 | O'Neill, 2010 | Journal of Reconstructive Microsurgery | UK | Cross-sectional | |  |  | Orthoplastic | Public | Absent | Multi-center | D1:0, D2:1, D3:1, D4:2, D5:2, D6:2 (Total: 8/26) | 30.77% |
| 37 | Morris, 2018 | European journal of orthopaedic surgery & traumatologie | UK | Prospective cohort | | 40 |  | Orthoplastic | Public | Absent | Single-center | D1:2, D2:2, D3:2, D4:1, D5:2, D6:0 (Total: 9/26) | 34.62% |
| 38 | Osinga, 2020 | Journal Of Bone And Joint Infection | Switzerland | Retrospective cohort | | 160 |  | Orthoplastic | Academic | Absent | Single-center | D1:2, D2:1, D3:1, D4:2, D5:2, D6:1 (Total: 9/26) | 34.62% |
| 39 | Habarth-Morales, 2024 | Journal of Reconstructive Microsurgery | USA | Retrospective cohort | | 1030 |  | Orthoplastic | Academic | Absent | Multi-center | D1:2, D2:1, D3:2, D4:1, D5:1, D6:2 (Total: 9/26) | 34.62% |
| 40 | Rayner, 2016 | Journal of Psychosomatic Research | UK | Cross-sectional | | 566 | 2011 | Orthoplastic | Academic | Absent | Single-center | D1:2, D2:2, D3:1, D4:3, D5:1, D6:0 (Total: 9/26) | 34.62% |
| 41 | Abu el Hawa (2), 2022 | Advances in Wound Care | USA | Retrospective cohort | | 62 |  | Orthoplastic | Academic | Absent | Single-center | D1:2, D2:1, D3:2, D4:2, D5:1, D6:1 (Total: 9/26) | 34.62% |
| 42 | Yamamoto, 2022 | Plastic and Reconstructive Surgery - Global Open | USA | Retrospective cohort | | 420 |  | Orthoplastic | Academic | Absent | Single-center | D1:1, D2:1, D3:1, D4:3, D5:2, D6:2 (Total: 10/26) | 38.46% |
| 43 | Sommar, 2015 | Journal of Trauma Management & Outcomes [Electronic Resource] | Sweden | Retrospective cohort | | 52 | 2008 | Orthoplastic | Academic | Absent | Single-center | D1:2, D2:4, D3:1, D4:1, D5:2, D6:0 (Total: 10/26) | 38.46% |
| 44 | Trickett, 2015 | Annals of the Royal College of Surgeons of UK | UK | Retrospective cohort | | 95 |  | Orthoplastic | Academic | Absent | Single-center | D1:0, D2:4, D3:2, D4:2, D5:2, D6:1 (Total: 11/26) | 42.31% |
| 45 | Seitz, 2017 | European Journal of Plastic Surgery | USA | Retrospective cohort | | 92 |  | Orthoplastic | Academic | Level 1 | Single-center | D1:0, D2:3, D3:1, D4:5, D5:2, D6:0 (Total: 11/26) | 42.31% |
| 46 | Azoury, 2021 | Journal of Reconstructive Microsurgery | USA | Review | |  |  | Orthoplastic | Academic | Absent | Single-center | D1:1, D2:4, D3:3, D4:1, D5:1, D6:1 (Total: 11/26) | 42.31% |
| 47 | Winstanley, 2022 | Bone & Joint Journal | UK | Prospective cohort | | 1175 |  | Orthoplastic | Public | Absent | Multi-center | D1:0, D2:4, D3:2, D4:1, D5:3, D6:2 (Total: 12/26) | 46.15% |
| 48 | Mehta, 2022 | European journal of orthopaedic surgery & traumatologie | UK | Retrospective cohort | | 135 |  | Orthoplastic | Academic | Absent | Single-center | D1:0, D2:4, D3:2, D4:2, D5:1, D6:3 (Total: 12/26) | 46.15% |
| 49 | Sargazi, 2015 | Journal of Plastic, Reconstructive & Aesthetic Surgery: JPRAS | UK | Retrospective cohort | | 18 | 2014 | Orthoplastic | Academic | Absent | Single-center | D1:0, D2:4, D3:2, D4:4, D5:2, D6:1 (Total: 13/26) | 50.00% |
| 50 | Rymer, 2017 | Injury | UK | Retrospective cohort | | 84 |  | Orthoplastic | Public | Absent | Multi-center | D1:2, D2:4, D3:1, D4:3, D5:2, D6:1 (Total: 13/26) | 50.00% |
| 51 | Khan, 2007 | Plastic & Reconstructive Surgery | UK; Australia | Retrospective cohort | | 24 |  | Orthoplastic | Academic | Absent | Single-center | D1:1, D2:3, D3:2, D4:2, D5:2, D6:3 (Total: 13/26) | 50.00% |
| 52 | Shahid, 2013 | Archives of Trauma Research | UK | Case series | | 12 | 2009 | Orthoplastic | Public | Absent | Single-center | D1:0, D2:4, D3:1, D4:2, D5:2, D6:4 (Total: 13/26) | 50.00% |
| 53 | Chummun, 2015 | Injury | UK | Retrospective cohort | | 68 | 2006 | Orthoplastic | Academic | Absent | Single-center | D1:1, D2:3, D3:3, D4:3, D5:1, D6:2 (Total: 13/26) | 50.00% |
| 54 | Boriani, 2017 | Journal of Plastic, Reconstructive & Aesthetic Surgery: JPRAS | UK; Italy; Pakistan | Prospective cohort | | 154 | 2006 | Orthoplastic | Public | Absent | Multi-center | D1:3, D2:3, D3:3, D4:2, D5:1, D6:1 (Total: 13/26) | 50.00% |
| 55 | Sobti, 2021 | Plastic and Reconstructive Surgery - Global Open | USA | Case series | | 65 | 2019 | Orthoplastic | Academic | Absent | Single-center | D1:1, D2:4, D3:2, D4:4, D5:1, D6:2 (Total: 14/26) | 53.85% |
| 56 | Chummun, 2011 | European Journal of Plastic Surgery | UK | Retrospective cohort | | 45 | 2010 | Orthoplastic | Academic | Absent | Single-center | D1:2, D2:4, D3:3, D4:3, D5:2, D6:0 (Total: 14/26) | 53.85% |
| 57 | Yarrow, 2015 | Annals of the Royal College of Surgeons of UK | UK | Prospective cohort | | 86 | 2009 | Orthoplastic | Public | Absent | Multi-center | D1:2, D2:4, D3:1, D4:5, D5:1, D6:2 (Total: 15/26) | 57.69% |
| 58 | Aljawadi, 2021 | Archives of Orthopaedic & Trauma Surgery | UK | Prospective cohort | | 120 |  | Orthoplastic | Academic | Absent | Single-center | D1:2, D2:3, D3:3, D4:2, D5:2, D6:3 (Total: 15/26) | 57.69% |
| 59 | Thng, 2024 | Journal of Wound Care | Singapore | Retrospective cohort | | 103 | 2019 | Orthoplastic | Public | Absent | Single-center | D1:2, D2:3, D3:2, D4:4, D5:1, D6:3 (Total: 15/26) | 57.69% |
| 60 | Cullen, 2024 | Acta Orthopaedica Belgica | UK | Retrospective cohort | | 85 |  | Orthoplastic | Public | Absent | Single-center | D1:2, D2:3, D3:3, D4:4, D5:2, D6:1 (Total: 15/26) | 57.69% |
| 61 | Naique, 2006 | Journal of Bone & Joint Surgery - British Volume | UK | Retrospective cohort | | 72 |  | Orthoplastic | Academic | Absent | Single-center | D1:1, D2:4, D3:2, D4:5, D5:2, D6:1 (Total: 15/26) | 57.69% |
| 62 | Chummun, 2013 | Plastic & Reconstructive Surgery | UK | Retrospective cohort | | 68 | 2006 | Orthoplastic | Academic | Absent | Single-center | D1:1, D2:4, D3:2, D4:4, D5:2, D6:2 (Total: 15/26) | 57.69% |
| 63 | Page, 2015 | Injury | UK | Retrospective cohort | | 101 |  | Orthoplastic | Public | Absent | Single-center | D1:0, D2:4, D3:2, D4:6, D5:2, D6:2 (Total: 16/26) | 61.54% |
| 64 | Higgin, 2021 | Injury | UK | Retrospective cohort | | 116 | 2012 | Orthoplastic | Academic | Level 1 | Single-center | D1:1, D2:3, D3:1, D4:6, D5:3, D6:2 (Total: 16/26) | 61.54% |
| 65 | Naga, 2021 | Plastic & Reconstructive Surgery | USA | Retrospective cohort | | 173 |  | Orthoplastic | Academic | Level 1 | Single-center | D1:2, D2:4, D3:2, D4:2, D5:2, D6:4 (Total: 16/26) | 61.54% |
| 66 | Cao, 2022 | Journal of Clinical Medicine | China | Retrospective cohort | | 394 |  | Orthoplastic | Academic | Absent | Single-center | D1:1, D2:4, D3:1, D4:5, D5:2, D6:3 (Total: 16/26) | 61.54% |
| 67 | Mathews, 2015 | Injury | UK | Retrospective cohort | | 73 |  | Orthoplastic | Public | Absent | Single-center | D1:2, D2:4, D3:2, D4:4, D5:2, D6:3 (Total: 17/26) | 65.38% |
| 68 | Hendrickson, 2020 | Injury | UK | Retrospective cohort | | 156 |  | Orthoplastic | Public | Level 1 | Single-center | D1:2, D2:4, D3:2, D4:4, D5:2, D6:3 (Total: 17/26) | 65.38% |
| 69 | Joosten, 2024 | European Journal of Trauma & Emergency Surgery | Netherlands | Review | | 520 |  | Orthoplastic |  | Level 1 | Multi-center | D1:1, D2:4, D3:2, D4:5, D5:2, D6:3 (Total: 17/26) | 65.38% |
| 70 | Lacey, 2024 | JPRAS Open | UK | Retrospective cohort | | 133 | 2020 | Orthoplastic | Academic | Level 1 | Single-center | D1:1, D2:3, D3:2, D4:4, D5:3, D6:4 (Total: 17/26) | 65.38% |
| 71 | Kim, 2023 | Journal of Clinical Medicine | USA | Retrospective cohort |  | |  | Vascular | Academic | Absent | Single-center | D1:1, D2:0, D3:0, D4:0, D5:0, D6:0 (Total: 1/26) | 3.85% |
| 72 | Patel, 2022 | Journal of Vascular Surgery | USA | Retrospective cohort | | 35591 |  | Vascular | Academic | Absent | Single-center | D1:1, D2:0, D3:1, D4:0, D5:1, D6:0 (Total: 3/26) | 11.54% |
| 73 | Goka, 2017 | Annals of Vascular Surgery | UK | Review | | 67445 |  | Vascular | Absent | Absent | Multi-center | D1:0, D2:0, D3:0, D4:0, D5:2, D6:2 (Total: 4/26) | 15.38% |
| 74 | Medhekar, 2017 | Journal of Vascular Surgery | USA | Retrospective cohort | | 49576 |  | Vascular | Absent | Absent | Multi-center | D1:0, D2:2, D3:0, D4:0, D5:1, D6:1 (Total: 4/26) | 15.38% |
| 75 | Ge, 2024 | International Wound Journal | Singapore | Other | | 406 | 2020 | Vascular | Public | Absent | Multi-center | D1:1, D2:1, D3:1, D4:0, D5:1, D6:0 (Total: 4/26) | 15.38% |
| 76 | O'Banion, 2024 | Journal of Vascular Surgery | USA | Review | |  |  | Vascular | Absent | Absent |  | D1:1, D2:0, D3:1, D4:0, D5:1, D6:1 (Total: 4/26) | 15.38% |
| 77 | Yamada, 2013 | International Journal of Vascular Medicine | Japan | Other | | 194 | 2010 | Vascular | Academic | Absent | Single-center | D1:1, D2:1, D3:2, D4:1, D5:1, D6:0 (Total: 6/26) | 23.08% |
| 78 | Lin, 2021 | BMC Family Practice | Taiwan | Retrospective cohort | | 668 |  | Vascular | Academic | Absent | Single-center | D1:2, D2:1, D3:1, D4:0, D5:1, D6:1 (Total: 6/26) | 23.08% |
| 79 | Sanguily, 2016 | Vascular Disease Management | USA | Retrospective cohort | | 500 | 2010 | Vascular | Public | Absent | Single-center | D1:1, D2:3, D3:1, D4:0, D5:1, D6:0 (Total: 6/26) | 23.08% |
| 80 | Schmidt, 2017 | Diabetes Research & Clinical Practice | USA | Retrospective cohort | | 3858 | 2006 | Vascular | Academic | Absent | Single-center | D1:1, D2:2, D3:2, D4:0, D5:1, D6:1 (Total: 7/26) | 26.92% |
| 81 | Salvo, 2017 | Current Treatment Options in Cardiovascular Medicine | USA | Other | |  |  | Vascular | Other | Absent | Single-center | D1:2, D2:1, D3:2, D4:0, D5:1, D6:1 (Total: 7/26) | 26.92% |
| 82 | Choke, 2020 | Diabetes/Metabolism Research Reviews | Singapore | Review | |  |  | Vascular | Public | Absent | Single-center | D1:3, D2:1, D3:2, D4:0, D5:0, D6:1 (Total: 7/26) | 26.92% |
| 83 | Prieto, 2020 | The Journal of Trauma and Acute Care Surgery | USA | Retrospective cohort | | 702 |  | Vascular | Other | Level 1;2 | Multi-center | D1:1, D2:3, D3:3, D4:0, D5:0, D6:0 (Total: 7/26) | 26.92% |
| 84 | Manu, 2014 | International Journal of Lower Extremity Wounds | UK | Retrospective cohort | | 597 | 1981 | Vascular | Academic | Absent | Single-center | D1:1, D2:1, D3:0, D4:0, D5:2, D6:3 (Total: 7/26) | 26.92% |
| 85 | Neville, 2017 | Blood Purification | USA | Other | |  |  | Vascular | Academic | Absent |  | D1:2, D2:1, D3:1, D4:0, D5:1, D6:2 (Total: 7/26) | 26.92% |
| 86 | Houghton, 2021 | Annals of Vascular Surgery | UK | Retrospective cohort | | 198 | 2018 | Vascular | Academic | Absent | Single-center | D1:1, D2:1, D3:1, D4:3, D5:2, D6:0 (Total: 8/26) | 30.77% |
| 87 | Kim, 2022 | Wounds-A Compendium of Clinical Research & Practice | USA | Review | |  |  | Vascular | Absent | Absent | Multi-center | D1:1, D2:3, D3:2, D4:1, D5:0, D6:1 (Total: 8/26) | 30.77% |
| 88 | Ge, 2024 | International Journal of Lower Extremity Wounds | Singapore | Prospective cohort | | 2798 | 2020 | Vascular | Public | Absent | Multi-center | D1:0, D2:1, D3:1, D4:3, D5:1, D6:2 (Total: 8/26) | 30.77% |
| 89 | Driver, 2005 | Diabetes Care | USA | Retrospective cohort | | 4940 | 1995 | Vascular | Military | Absent | Single-center | D1:2, D2:2, D3:2, D4:0, D5:1, D6:1 (Total: 8/26) | 30.77% |
| 90 | Ramanan, 2017 | Journal of Vascular Surgery | USA | Retrospective cohort | | 128 |  | Vascular | Academic | Absent | Single-center | D1:2, D2:2, D3:1, D4:1, D5:1, D6:1 (Total: 8/26) | 30.77% |
| 91 | Flores, 2019 | Journal of Vascular Surgery | USA | Retrospective cohort | | 909 | 2013 | Vascular | Academic | Absent | Single-center | D1:2, D2:1, D3:2, D4:1, D5:1, D6:2 (Total: 9/26) | 34.62% |
| 92 | Hemingway, 2021 | Annals of Vascular Surgery | USA | Retrospective cohort | | 609 | 2014 | Vascular | Academic | Level 1 | Single-center | D1:1, D2:1, D3:1, D4:3, D5:2, D6:1 (Total: 9/26) | 34.62% |
| 93 | Khetarpaul, 2022 | Frontiers in Rehabilitation Sciences | USA | Review | |  |  | Vascular | Other | Absent |  | D1:2, D2:2, D3:2, D4:1, D5:2, D6:0 (Total: 9/26) | 34.62% |
| 94 | Campbell, 2023 | Journal of Vascular Surgery | USA | Retrospective cohort | | 220 |  | Vascular | Academic | Absent | Single-center | D1:3, D2:1, D3:2, D4:2, D5:1, D6:0 (Total: 9/26) | 34.62% |
| 95 | Lew, 2023 | International Journal of Lower Extremity Wounds | USA | Retrospective cohort | | 337 | 2015 | Vascular | Academic | Absent | Single-center | D1:2, D2:3, D3:1, D4:2, D5:1, D6:0 (Total: 9/26) | 34.62% |
| 96 | Tan, 2010 | Biomedical Imaging & Intervention Journal | Singapore | Retrospective cohort | | 42 | 2001 | Vascular | Public | Absent | Single-center | D1:3, D2:3, D3:1, D4:1, D5:1, D6:0 (Total: 9/26) | 34.62% |
| 97 | Williams, 2012 | Annals of Vascular Surgery | UK | Other | |  | 2006 | Vascular | Public | Absent | Single-center | D1:1, D2:2, D3:1, D4:2, D5:1, D6:2 (Total: 9/26) | 34.62% |
| 98 | Armstrong, 2012 | Diabetes/Metabolism Research Reviews | USA | Retrospective cohort | | 374 | 2008 | Vascular | Academic | Absent | Single-center | D1:2, D2:1, D3:1, D4:3, D5:1, D6:1 (Total: 9/26) | 34.62% |
| 99 | Khan, 2018 | Diabetic Foot & Ankle | USA | Other | |  |  | Vascular | Academic | Absent |  | D1:3, D2:1, D3:1, D4:2, D5:1, D6:1 (Total: 9/26) | 34.62% |
| 100 | Roberts, 2022 | International Wound Journal | Canada | Prospective cohort | | 162 | 2017 | Vascular | Academic | Absent | Single-center | D1:2, D2:1, D3:2, D4:1, D5:1, D6:2 (Total: 9/26) | 34.62% |
| 101 | Deery, 2022 | Journal of Vascular Surgery | USA | Retrospective cohort | | 131 | 2012 | Vascular | Academic | Absent | Single-center | D1:2, D2:1, D3:3, D4:1, D5:1, D6:1 (Total: 9/26) | 34.62% |
| 102 | Houghton, 2024 | European Journal of Vascular & Endovascular Surgery | UK | Other | | 928 | 2018 | Vascular | Academic | Absent | Single-center | D1:2, D2:2, D3:3, D4:0, D5:1, D6:2 (Total: 10/26) | 38.46% |
| 103 | Bazikian, 2024 | Journal of Foot & Ankle Research | USA | Retrospective cohort | | 731 | 2018 | Vascular | Public | Absent | Single-center | D1:3, D2:2, D3:1, D4:3, D5:1, D6:0 (Total: 10/26) | 38.46% |
| 104 | Robinson, 2017 | Journal of Vascular Surgery | USA | Retrospective cohort | | 257 |  | Vascular | Academic | Absent | Single-center | D1:1, D2:2, D3:1, D4:2, D5:1, D6:3 (Total: 10/26) | 38.46% |
| 105 | Williams, 2018 | Diabetes Research and Clinical Practice | UK | Other | | 333 | 2006 | Vascular | Public | Absent | Single-center | D1:3, D2:1, D3:2, D4:1, D5:2, D6:1 (Total: 10/26) | 38.46% |
| 106 | Houghton, 2019 | BMJ Open | UK | Retrospective cohort | | 420 |  | Vascular | Academic | Absent | Single-center | D1:3, D2:1, D3:2, D4:2, D5:3, D6:0 (Total: 11/26) | 42.31% |
| 107 | Gabel, 2020 | Journal of Vascular Surgery | USA | Retrospective cohort | | 236 |  | Vascular | Other | Absent | Single-center | D1:2, D2:0, D3:2, D4:4, D5:3, D6:0 (Total: 11/26) | 42.31% |
| 108 | Campbell, 2024 | Journal of Vascular Surgery | USA | Other | | 871 |  | Vascular | Academic | Absent | Single-center | D1:2, D2:3, D3:1, D4:2, D5:2, D6:1 (Total: 11/26) | 42.31% |
| 109 | Gordon, 1997 | Seminars in Perioperative Nursing | USA | Retrospective cohort | | 33 | 1996 | Vascular | Academic | Absent | Single-center | D1:3, D2:1, D3:3, D4:1, D5:1, D6:2 (Total: 11/26) | 42.31% |
| 110 | Zayed, 2009 | International Journal of Clinical Practice | UK | Retrospective cohort | | 312 | 2003 | Vascular | Academic | Absent | Single-center | D1:1, D2:1, D3:2, D4:3, D5:2, D6:2 (Total: 11/26) | 42.31% |
| 111 | Casey, 2015 | Annals of Vascular Surgery | USA | Retrospective cohort | | 16 | 2011 | Vascular | Military | Absent | Single-center | D1:3, D2:1, D3:1, D4:2, D5:2, D6:2 (Total: 11/26) | 42.31% |
| 112 | Nickinson, 2020 | Diabetes/Metabolism Research Reviews | UK | Review | | 4010 |  | Vascular | Multiple | Absent | Multi-center | D1:3, D2:1, D3:2, D4:3, D5:2, D6:1 (Total: 12/26) | 46.15% |
| 113 | Fereydooni, 2022 | Annals of Vascular Surgery | USA | Retrospective cohort | | 63 | 2018 | Vascular | Academic | Absent | Single-center | D1:3, D2:2, D3:1, D4:2, D5:2, D6:2 (Total: 12/26) | 46.15% |
| 114 | Attinger, 2008 | Gynecologic Oncology | USA | Other | |  | 1999 | Vascular | Academic | Absent | Single-center | D1:3, D2:4, D3:2, D4:3, D5:0, D6:0 (Total: 12/26) | 46.15% |
| 115 | Eggert, 2016 | Undersea & Hyperbaric Medicine | USA | Retrospective cohort | | 159 | 2005 | Vascular | Public | Absent | Single-center | D1:2, D2:3, D3:2, D4:2, D5:3, D6:1 (Total: 13/26) | 50.00% |
| 116 | Nickinson, 2021 | European Journal of Vascular & Endovascular Surgery | UK | Retrospective cohort | | 566 | 2018 | Vascular | Academic | Absent | Single-center | D1:2, D2:2, D3:3, D4:3, D5:1, D6:2 (Total: 13/26) | 50.00% |
| 117 | Martinez-Singh, 2022 | Seminars in Vascular Surgery | USA | Review | |  |  | Vascular | Academic | Absent | Single-center | D1:3, D2:3, D3:1, D4:2, D5:1, D6:3 (Total: 13/26) | 50.00% |
| 118 | Brumberg, 2021 | Annals of Vascular Surgery | USA | Retrospective cohort | | 10 | 2016 | Vascular | Public | Level 2 | Single-center | D1:1, D2:3, D3:3, D4:5, D5:2, D6:0 (Total: 14/26) | 53.85% |

***Supplement 1.*** Complete Study Characteristics and Domain Score Profiles for All Included Limb Salvage Center Studies
Comprehensive individual study data for all 118 included studies showing detailed breakdown by center type, geographic distribution, methodological characteristics, and complete domain scoring (D1-D6). Data demonstrates wide performance variation within center types (3.85%-69.23% total scores) and enables granular analysis of center-specific patterns across organizational domains. Domain score transparency supports reproducibility and enables identification of high-performing centers for benchmarking purposes.

*Abbreviations: N, Sample;*
