# Supplementary figures and images for: Defining orthoplastic limb salvage centers: a systematic review
Source: Arch Orthop Trauma Surg. 2026 May 2;146(1):171. doi: 10.1007/s00402-026-06325-0 (PMC13135554; doi:10.1007/s00402-026-06325-0)

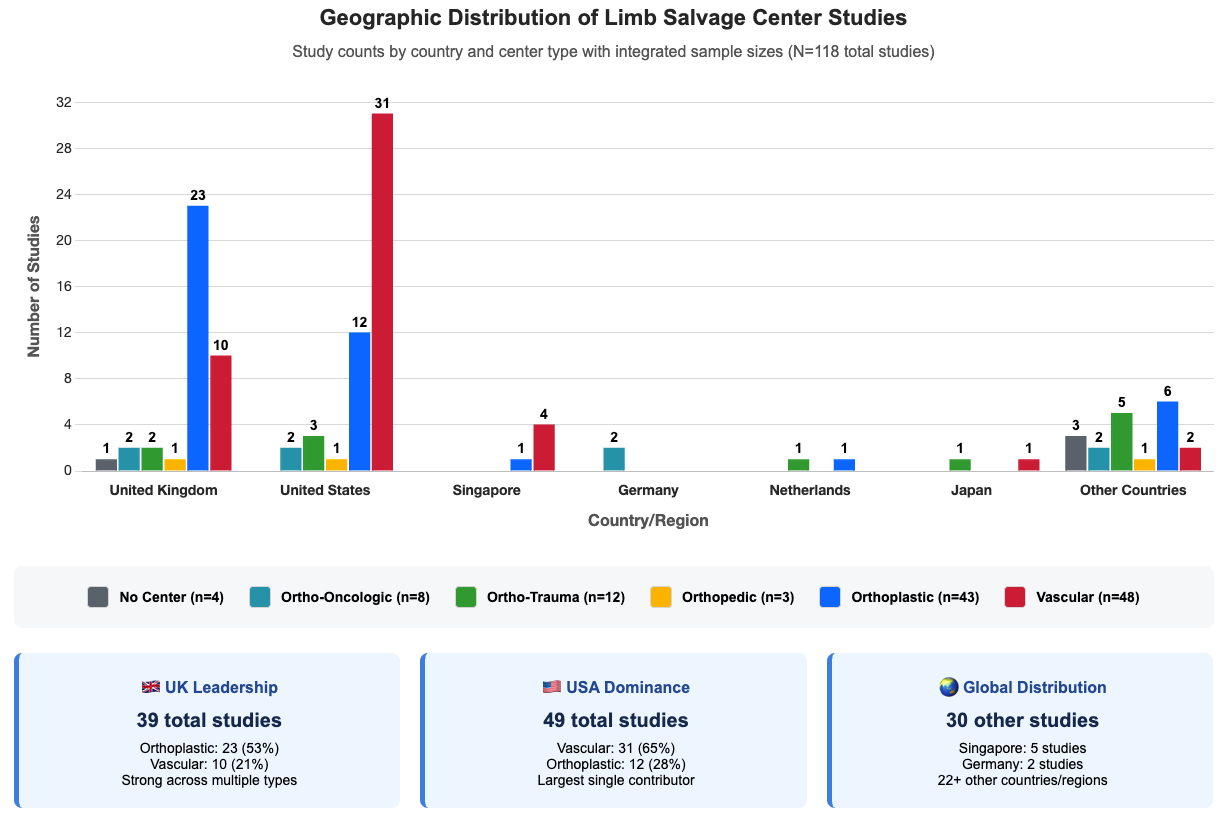

Supplement: Supplementary file 3 — Supplement 2. Geographic distribution of limb salvage center studies by country and center type. Global distribution of 118 studies across 26 countries showing clear geographic specialization patterns. USA dominates vascular research (65% of global vascular literature; 31/48 studies) while UK leads orthoplastic research (53% of global orthoplastic literature; 23/43 studies), indicating healthcare system-influenced practice development with emerging contributions from 22 additional countries. [file 402_2026_6325_MOESM3_ESM.png]

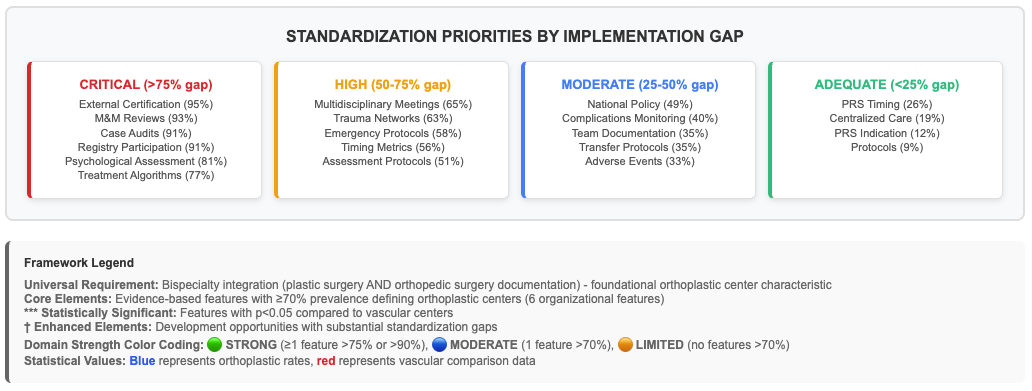

Supplement: Supplementary file 5 — Supplement 4. Orthoplastic center standardization priorities. Standardization priorities for orthoplastic center organizational features ranked by implementation gap. CRITICAL priorities (red, >75% gap) require immediate consensus development; HIGH priorities (orange, 50–75% gap) need structured standardization; MODERATE priorities (blue, 25–50% gap) require refinement of existing practices; ADEQUATE priorities (green, <25% gap) need validation and specification only. Gap percentages represent the difference between current reporting rates and optimal 100% implementation. [file 402_2026_6325_MOESM5_ESM.png]
