# Supplementary material for: Defining orthoplastic limb salvage centers: a systematic review
Source: Arch Orthop Trauma Surg. 2026 May 2;146(1):171. doi: 10.1007/s00402-026-06325-0 (PMC13135554; doi:10.1007/s00402-026-06325-0)
Supplement: Supplementary file 4 — Supplement 3. Risk of bias assessment. Risk of bias assessment using the appropriate validated tools across studies. While most studies demonstrated low to moderate risk in outcome measurement and reporting, common limitations included retrospective designs, inconsistent outcome protocols, and potential selection bias in single-center studies. [file 402_2026_6325_MOESM4_ESM.docx]

**Supplement 3 - Comprehensive Risk of Bias Assessment**

| **Type of Center** | **Study** | **Assessment Tool** | **Domain Scores/Results** | **Total Score** | **Summary/Notes** |
| --- | --- | --- | --- | --- | --- |
| Orthoplastic | Aljawadi, 2021 | MINORS (non-comparative) | D1:2, D2:1, D3:2, D4:2, D5:1, D6:2, D7:2, D8:1 | 13/16 |  |
| Vascular | Brumberg, 2021 | MINORS (non-comparative) | D1:2, D2:2, D3:1, D4:2, D5:1, D6:2, D7:1, D8:0 | 11/16 |  |
| Vascular | Casey, 2015 | MINORS (non-comparative) | D1:2, D2:1, D3:0, D4:2, D5:1, D6:2, D7:2, D8:0 | 10/16 |  |
| Vascular | Deery, 2022 | MINORS (non-comparative) | D1:2, D2:2, D3:2, D4:2, D5:1, D6:2, D7:1, D8:1 | 13/16 |  |
| Vascular | Driver, 2005 | MINORS (non-comparative) | D1:2, D2:1, D3:1, D4:2, D5:1, D6:2, D7:1, D8:1 | 11/16 |  |
| Vascular | Fereydooni, 2022 | MINORS (non-comparative) | D1:2, D2:2, D3:1, D4:2, D5:1, D6:2, D7:1, D8:0 | 11/16 |  |
| Vascular | Gordon, 1997 | MINORS (non-comparative) | D1:2, D2:1, D3:1, D4:2, D5:1, D6:2, D7:1, D8:0 | 10/16 |  |
| Orthoplastic | Hadfield, 2022 | MINORS (non-comparative) | D1:2, D2:2, D3:2, D4:2, D5:0, D6:2, D7:2, D8:0 | 14/16 |  |
| Orthoplastic | Higgin, 2021 | MINORS (non-comparative) | D1:2, D2:2, D3:0, D4:2, D5:1, D6:2, D7:2, D8:1 | 12/16 |  |
| Ortho-Trauma | Hohmann, 2017 | MINORS (non-comparative) | D1:2, D2:2, D3:0, D4:2, D5:1, D6:2, D7:2, D8:1 | 12/16 |  |
| Orthopedic | Hruby, 2017 | MINORS (non-comparative) | D1:2, D2:1, D3:0, D4:2, D5:0, D6:2, D7:2, D8:0 | 11/16 |  |
| Ortho-trauma | Izawa, 2024 | MINORS (non-comparative) | D1:2, D2:2, D3:0, D4:2, D5:1, D6:2, D7:2, D8:0 | 11/16 |  |
| Vascular | Kim, 2023 | MINORS (non-comparative) | D1:1, D2:2, D3:1, D4:2, D5:1, D6:1, D7:0, D8:0 | 8/16 |  |
| Orthoplastic | Lacey, 2024 | MINORS (non-comparative) | D1:2, D2:2, D3:1, D4:2, D5:1, D6:2, D7:2, D8:0 | 14/16 |  |
| Ortho-Oncologic | Leckenby, 2017 | MINORS (non-comparative) | D1:2, D2:2, D3:1, D4:2, D5:1, D6:2, D7:1, D8:0 | 15/16 |  |
| Vascular | Manu, 2014 | MINORS (non-comparative) | D1:1, D2:2, D3:2, D4:2, D5:1, D6:2, D7:1, D8:0 | 13/16 |  |
| Ortho-trauma | Messner, 2020 | MINORS (non-comparative) | D1:1, D2:2, D3:0, D4:2, D5:1, D6:2, D7:2, D8:0 | 12/16 |  |
| Orthoplastic | Morris, 2018 | MINORS (non-comparative) | D1:2, D2:2, D3:2, D4:2, D5:2, D6:2, D7:2, D8:0 | 14/16 |  |
| Orthoplastic | Osinga, 2020 | MINORS (non-comparative) | D1:1, D2:0, D3:0, D4:2, D5:1, D6:1, D7:2, D8:0 | 7/16 |  |
| Vascular | Patel, 2022 | MINORS (non-comparative) | D1:1, D2:1, D3:1, D4:2, D5:1, D6:2, D7:0, D8:0 | 8/16 |  |
| Vascular | Ramanan, 2017 | MINORS (non-comparative) | D1:2, D2:2, D3:1, D4:2, D5:1, D6:2, D7:1, D8:1 | 12/16 |  |
| Orthoplastic | Rayner, 2016 | MINORS (non-comparative) | D1:2, D2:1, D3:1, D4:2, D5:1, D6:1, D7:2, D8:0 | 10/16 |  |
| Vascular | Roberts, 2022 | MINORS (non-comparative) | D1:2, D2:2, D3:1, D4:2, D5:1, D6:2, D7:1, D8:0 | 11/16 |  |
| Ortho-trauma | Rozbruch, 2015 | MINORS (non-comparative) | D1:2, D2:2, D3:0, D4:2, D5:1, D6:1, D7:2, D8:0 | 10/16 |  |
| Vascular | Sanguily, 2016 | MINORS (non-comparative) | D1:2, D2:1, D3:1, D4:2, D5:1, D6:2, D7:1, D8:1 | 11/16 |  |
| Orthoplastic | Sayyed, 2024 | MINORS (non-comparative) | D1:2, D2:2, D3:1, D4:2, D5:1, D6:1, D7:2, D8:0 | 11/16 |  |
| Orthoplastic | Shahid, 2013 | MINORS (non-comparative) | D1:2, D2:1, D3:0, D4:2, D5:1, D6:2, D7:2, D8:0 | 10/16 |  |
| Orthoplastic | Sobti, 2021 | MINORS (non-comparative) | D1:1, D2:0, D3:2, D4:1, D5:0, D6:1, D7:2, D8:0 | 7/16 |  |
| Vascular | Tan, 2010 | MINORS (non-comparative) | D1:2, D2:2, D3:1, D4:2, D5:1, D6:2, D7:1, D8:0 | 11/16 |  |
| Orthoplastic | Winstanley, 2022 | MINORS (non-comparative) | D1:2, D2:2, D3:2, D4:2, D5:2, D6:2, D7:2, D8:1 | 15/16 |  |
| Vascular | Yamada, 2013 | MINORS (non-comparative) | D1:2, D2:1, D3:1, D4:2, D5:1, D6:2, D7:1, D8:0 | 10/16 |  |
| Orthoplastic | Yarrow, 2015 | MINORS (non-comparative) | D1:2, D2:2, D3:2, D4:2, D5:1, D6:1, D7:2, D8:1 | 13/16 |  |
| Vascular | Zayed, 2009 | MINORS (non-comparative) | D1:2, D2:1, D3:1, D4:2, D5:1, D6:2, D7:1, D8:1 | 11/16 |  |
| Ortho-Oncologic | Zeller, 2019 | MINORS (non-comparative) | D1:2, D2:2, D3:1, D4:2, D5:1, D6:2, D7:1, D8:0 | 15/16 |  |
| Ortho-Oncologic | Abarca, 2018 | MINORS (comparative) | D1:2, D2:2, D3:1, D4:2, D5:1, D6:2, D7:1, D8:1, D9:2, D10:2, D11:2, D12:2 | 20/24 |  |
| Vascular | Bazikian, 2024 | MINORS (comparative) | D1:2, D2:1, D3:1, D4:2, D5:1, D6:2, D7:2, D8:1, D9:2, D10:2, D11:1, D12:2 | 19/24 |  |
| No Center | Berner, 2024 | MINORS (comparative) | D1:2, D2:0, D3:2, D4:2, D5:1, D6:2, D7:2, D8:0, D9:1, D10:2, D11:1, D12:0 | 15/24 |  |
| Orthoplastic | Boriani, 2017 | MINORS (comparative) | D1:2, D2:2, D3:2, D4:2, D5:1, D6:2, D7:1, D8:1, D9:2, D10:2, D11:1, D12:2 | 20/24 |  |
| Ortho-trauma | Bunn, 2021 | MINORS (comparative) | D1:2, D2:2, D3:1, D4:2, D5:1, D6:2, D7:1, D8:1, D9:2, D10:2, D11:2, D12:2 | 20/24 |  |
| Vascular | Campbell, 2024 | MINORS (comparative) | D1:2, D2:2, D3:1, D4:2, D5:1, D6:2, D7:1, D8:1, D9:2, D10:2, D11:2, D12:2 | 20/24 |  |
| Vascular | Campbell, 2023 | MINORS (comparative) | D1:2, D2:1, D3:1, D4:2, D5:1, D6:2, D7:2, D8:1, D9:2, D10:2, D11:1, D12:2 | 19/24 |  |
| Orthoplastic | Cao, 2022 | MINORS (comparative) | D1:2, D2:1, D3:1, D4:2, D5:1, D6:2, D7:1, D8:1, D9:2, D10:2, D11:2, D12:2 | 19/24 |  |
| Orthoplastic | Chummun, 2011 | MINORS (comparative) | D1:2, D2:1, D3:1, D4:2, D5:1, D6:2, D7:1, D8:1, D9:1, D10:2, D11:1, D12:2 | 17/24 |  |
| Orthoplastic | Chummun, 2015 | MINORS (comparative) | D1:2, D2:2, D3:0, D4:2, D5:1, D6:2, D7:2, D8:0, D9:2, D10:2, D11:1, D12:2 | 18/24 |  |
| Orthoplastic | Cullen, 2024 | MINORS (comparative) | D1:2, D2:1, D3:1, D4:2, D5:1, D6:2, D7:1, D8:1, D9:2, D10:2, D11:1, D12:2 | 18/24 |  |
| Vascular | Eggert, 2016 | MINORS (comparative) | D1:2, D2:1, D3:1, D4:2, D5:1, D6:2, D7:2, D8:1, D9:1, D10:1, D11:1, D12:2 | 17/24 |  |
| Vascular | Flores, 2019 | MINORS (comparative) | D1:2, D2:1, D3:2, D4:2, D5:1, D6:2, D7:2, D8:1, D9:2, D10:2, D11:2, D12:2 | 23/24 |  |
| Vascular | Gabel, 2020 | MINORS (comparative) | D1:2, D2:1, D3:2, D4:2, D5:1, D6:2, D7:2, D8:1, D9:2, D10:2, D11:2, D12:2 | 23/24 |  |
| Ortho-Oncologic | Gaston, 2014 | MINORS (comparative) | D1:2, D2:2, D3:0, D4:2, D5:1, D6:2, D7:1, D8:1, D9:2, D10:2, D11:1, D12:2 | 18/24 |  |
| Vascular | Ge, 2024 | MINORS (comparative) | D1:2, D2:1, D3:1, D4:2, D5:1, D6:2, D7:2, D8:1, D9:2, D10:2, D11:1, D12:2 | 19/24 |  |
| Orthoplastic | Habarth-Morales, 2024 | MINORS (comparative) | D1:2, D2:1, D3:1, D4:2, D5:1, D6:2, D7:1, D8:2, D9:2, D10:2, D11:2, D12:2 | 20/24 |  |
| Vascular | Hemingway, 2021 | MINORS (comparative) | D1:2, D2:2, D3:1, D4:2, D5:1, D6:2, D7:1, D8:0, D9:2, D10:2, D11:1, D12:2 | 18/24 |  |
| Orthoplastic | Hendrickson, 2020 | MINORS (comparative) | D1:2, D2:2, D3:1, D4:2, D5:1, D6:2, D7:1, D8:1, D9:2, D10:2, D11:1, D12:2 | 19/24 |  |
| Vascular | Houghton, 2024 | MINORS (comparative) | D1:2, D2:1, D3:2, D4:2, D5:1, D6:2, D7:2, D8:1, D9:2, D10:2, D11:1, D12:2 | 20/24 |  |
| Ortho-trauma | Khadim, 2019 | MINORS (comparative) | D1:2, D2:1, D3:1, D4:2, D5:1, D6:2, D7:1, D8:1, D9:1, D10:2, D11:1, D12:2 | 17/24 |  |
| Orthoplastic | Khan, 2007 | MINORS (comparative) | D1:2, D2:1, D3:1, D4:2, D5:1, D6:2, D7:1, D8:1, D9:1, D10:2, D11:1, D12:2 | 17/24 |  |
| Ortho-trauma | Kim, 2022 | MINORS (comparative) | D1:2, D2:2, D3:1, D4:2, D5:1, D6:2, D7:1, D8:1, D9:2, D10:2, D11:2, D12:2 | 20/24 |  |
| Vascular | Lew, 2023 | MINORS (comparative) | D1:2, D2:1, D3:1, D4:2, D5:1, D6:2, D7:2, D8:1, D9:2, D10:2, D11:1, D12:2 | 19/24 |  |
| Vascular | Lin, 2021 | MINORS (comparative) | D1:2, D2:1, D3:1, D4:2, D5:1, D6:2, D7:2, D8:1, D9:2, D10:1, D11:1, D12:2 | 18/24 |  |
| Ortho-Oncologic | Malik, 2020 | MINORS (comparative) | D1:2, D2:2, D3:0, D4:2, D5:2, D6:2, D7:2, D8:0, D9:2, D10:2, D11:1, D12:2 | 19/24 |  |
| Orthoplastic | Mathews, 2015 | MINORS (comparative) | D1:2, D2:1, D3:1, D4:2, D5:1, D6:2, D7:1, D8:1, D9:1, D10:2, D11:1, D12:2 | 17/24 |  |
| No center | McCulloch, 2015 | MINORS (comparative) | D1:2, D2:2, D3:2, D4:2, D5:2, D6:2, D7:1, D8:1, D9:2, D10:2, D11:1, D12:2 | 21/24 |  |
| Vascular | Medhekar, 2017 | MINORS (comparative) | D1:2, D2:2, D3:0, D4:2, D5:1, D6:2, D7:2, D8:0, D9:2, D10:2, D11:1, D12:2 | 18/24 |  |
| Orthoplastic | Mehta, 2022 | MINORS (comparative) | D1:2, D2:2, D3:1, D4:2, D5:1, D6:2, D7:1, D8:1, D9:2, D10:2, D11:2, D12:2 | 20/24 |  |
| Orthoplastic | Naga, 2021 | MINORS (comparative) | D1:1, D2:1, D3:1, D4:2, D5:1, D6:2, D7:1, D8:0, D9:1, D10:2, D11:1, D12:2 | 15/24 |  |
| Orthoplastic | Naique, 2006 | MINORS (comparative) | D1:2, D2:2, D3:0, D4:2, D5:1, D6:2, D7:2, D8:0, D9:2, D10:2, D11:1, D12:2 | 18/24 |  |
| Vascular | Nickinson, 2021 | MINORS (comparative) | D1:2, D2:2, D3:1, D4:2, D5:2, D6:2, D7:1, D8:1, D9:2, D10:2, D11:2, D12:2 | 21/24 |  |
| Orthoplastic | Page, 2015 | MINORS (comparative) | D1:2, D2:2, D3:1, D4:2, D5:2, D6:2, D7:1, D8:1, D9:2, D10:2, D11:1, D12:2 | 19/24 |  |
| Ortho-trauma | Pari, 2021 | MINORS (comparative) | D1:2, D2:2, D3:0, D4:2, D5:1, D6:2, D7:1, D8:0, D9:2, D10:1, D11:1, D12:2 | 16/24 |  |
| Orthopedic | Pinzur, 2009 | MINORS (comparative) | D1:2, D2:1, D3:2, D4:2, D5:1, D6:2, D7:1, D8:2, D9:2, D10:2, D11:1, D12:2 | 20/24 |  |
| Vascular | Prieto, 2020 | MINORS (comparative) | D1:2, D2:2, D3:0, D4:2, D5:2, D6:1, D7:2, D8:0, D9:2, D10:2, D11:1, D12:2 | 18/24 |  |
| Orthoplastic | Rymer, 2017 | MINORS (comparative) | D1:2, D2:1, D3:0, D4:2, D5:1, D6:2, D7:1, D8:0, D9:1, D10:2, D11:2, D12:1 | 15/24 |  |
| Orthoplastic | Sargazi, 2015 | MINORS (comparative) | D1:2, D2:1, D3:1, D4:2, D5:1, D6:1, D7:1, D8:0, D9:1, D10:1, D11:1, D12:1 | 13/24 |  |
| Vascular | Schmidt, 2017 | MINORS (comparative) | D1:2, D2:1, D3:1, D4:2, D5:1, D6:2, D7:2, D8:1, D9:2, D10:1, D11:1, D12:2 | 18/24 |  |
| Orthoplastic | Sharif-Askary, 2021 | MINORS (comparative) | D1:2, D2:2, D3:1, D4:2, D5:1, D6:2, D7:1, D8:1, D9:1, D10:1, D11:2, D12:2 | 18/24 |  |
| Orthoplastic | Sommar, 2015 | MINORS (comparative) | D1:2, D2:2, D3:1, D4:2, D5:1, D6:2, D7:1, D8:1, D9:2, D10:2, D11:1, D12:2 | 19/24 |  |
| Orthoplastic | Thng, 2024 | MINORS (comparative) | D1:2, D2:1, D3:2, D4:2, D5:1, D6:2, D7:1, D8:1, D9:2, D10:2, D11:2, D12:2 | 20/24 |  |
| Orthoplastic | Trickett, 2015 | MINORS (comparative) | D1:2, D2:1, D3:1, D4:2, D5:1, D6:2, D7:1, D8:1, D9:2, D10:2, D11:1, D12:2 | 18/24 |  |
| Vascular | Williams, 2012 | MINORS (comparative) | D1:2, D2:2, D3:1, D4:2, D5:1, D6:2, D7:1, D8:0, D9:2, D10:2, D11:1, D12:2 | 18/24 |  |
| Vascular | Attinger et al., 2008 | SANRA | I1:2, I2:2, I3:0, I4:1, I5:1, I6:1 | 7/12 | Strong justification for topic importance, citing $20 billion annual cost of chronic wounds. Clear aims regarding Georgetown model viability. Comprehensive referencing with 26 citations. No literature search methodology described. Generally sound scientific reasoning but lacked critical analysis of evidence quality. |
| Orthoplastic | Azoury et al., 2021 | SANRA | I1:2, I2:2, I3:0, I4:2, I5:2, I6:1 | 9/12 | Accurately justified orthoplastic surgery importance for limb salvage. Clear aims regarding historical milestones and current principles. Comprehensive referencing with 58 citations. No literature search methodology described. Effective evidence-based approaches presentation. |
| Ortho-Trauma | Baus et al., 2020 | SANRA | I1:2, I2:2, I3:0, I4:1, I5:1, I6:1 | 7/12 | Moderate justification for war-related lower extremity injuries assessment. Well-defined objectives for 6/7/8/9 reconstruction doctrine review. Adequately referenced with 57 citations. Lack of described search strategy affects reproducibility. |
| Orthoplastic | Bridgeman & Crist, 2022 | SANRA | I1:2, I2:1, I3:0, I4:1, I5:1, I6:1 | 6/12 | Moderate justification for multidisciplinary limb preservation approaches. Well-defined aim for decision-making process review. Limited referencing with only 10 citations. No literature search methodology described increases selection bias risk. |
| Vascular | Choke et al., 2020 | SANRA | I1:2, I2:1, I3:0, I4:2, I5:2, I6:1 | 8/12 | Clear justification of diabetic limb-threatening ischaemia clinical importance. Well-defined comprehensive scope covering treatments. Appropriate and current referencing. Complete failure to describe literature search methodology. |
| Vascular | Khan et al., 2018 | SANRA | I1:2, I2:2, I3:0, I4:2, I5:1, I6:1 | 8/12 | Accurate justification of diabetic limb preservation clinical importance. Well-defined aims for four essential program components. Comprehensive referencing and logical framework. Complete failure to describe literature search methodology. |
| Vascular | Khetarpaul et al., 2022 | SANRA | I1:2, I2:2, I3:0, I4:2, I5:1, I6:1 | 8/12 | Clear justification using socioecological model framework. Well-defined aims for comprehensive program design. Extensive current referencing. Complete failure to describe literature search methodology. |
| Vascular | Kim et al., 2022 | SANRA | I1:2, I2:2, I3:0, I4:2, I5:1, I6:1 | 8/12 | Clear justification applying Donabedian model for quality improvement. Well-defined aims for collaborative initiatives. Comprehensive referencing with logical presentation. Complete failure to describe literature search methodology. |
| No center | Langer, 2014 | SANRA | I1:2, I2:2, I3:0, I4:2, I5:1, I6:1 | 8/12 | Clear justification of major limb injury management importance. Well-defined aims for limb salvage versus amputation decisions. Extensive referencing with logical presentation. Complete failure to describe literature search methodology. |
| Vascular | Martinez-Singh K et al., 2022 | SANRA | I1:2, I2:2, I3:2, I4:2, I5:1, I6:1 | 10/12 | Clear justification by establishing high CLTI mortality rates. Well-defined aims for four-step program construction. Comprehensive referencing with excellent logical presentation. Complete failure to describe literature search methodology. |
| Ortho-Oncologic | Morris et al., 2020 | SANRA | I1:2, I2:2, I3:0, I4:2, I5:1, I6:1 | 8/12 | Clear justification of limb salvage importance in austere environments. Well-defined aims with illustrative cases. Comprehensive referencing with logical presentation. Complete failure to describe literature search methodology. |
| Ortho-Trauma | Mullis et al., 2017 | SANRA | I1:2, I2:2, I3:0, I4:1, I5:2, I6:2 | 9/12 | Clear justification of combined orthopaedic and vascular injuries importance. Well-defined aims emphasizing team coordination. Appropriate referencing with excellent logical presentation. Complete failure to describe literature search methodology. |
| Vascular | Neville & Kayssi, 2017 | SANRA | I1:2, I2:2, I3:0, I4:2, I5:2, I6:1 | 9/12 | Clear justification by establishing significant mortality and economic burden. Well-defined aims for multidisciplinary program development. Appropriate referencing with excellent logical presentation. Complete failure to describe literature search methodology. |
| Vascular | Salvo et al., 2017 | SANRA | I1:2, I2:2, I3:0, I4:2, I5:2, I6:2 | 10/12 | Clear justification by establishing significant amputation rates and mortality. Well-defined aims for comprehensive literature presentation. Extensive referencing with excellent logical presentation. Complete failure to describe literature search methodology. |
| Orthopedic | Bond et al., 1999 | JBI Cross-sectional | Q1:Yes, Q2:Yes, Q3:Yes, Q4:Yes, Q5:Yes, Q6:No, Q7:Yes, Q8:Yes | 7/8 |  |
| Orthoplastic | Fan et al., 2021 | JBI Cross-sectional | Q1:Yes, Q2:Yes, Q3:Yes, Q4:Yes, Q5:Yes, Q6:Yes, Q7:Yes, Q8:Yes | 8/8 |  |
| Vascular | Houghton et al., 2021 | JBI Cross-sectional | Q1:Yes, Q2:Yes, Q3:Yes, Q4:Yes, Q5:Yes, Q6:Yes, Q7:Yes, Q8:Yes | 8/8 |  |
| Ortho-Trauma | Noorlander-Borgdorff et al., 2024 | JBI Cross-sectional | Q1:Yes, Q2:Yes, Q3:Yes, Q4:Yes, Q5:Yes, Q6:No, Q7:Yes, Q8:Yes | 7/8 |  |
| Orthoplastic | O'Neill et al., 2010 | JBI Cross-sectional | Q1:Yes, Q2:Yes, Q3:No, Q4:Unclear, Q5:Unclear, Q6:Unclear, Q7:Unclear, Q8:No | 2/8 |  |
| Orthoplastic | Radotra et al., 2022 | JBI Cross-sectional | Q1:Yes, Q2:Yes, Q3:No, Q4:Unclear, Q5:Unclear, Q6:Unclear, Q7:Unclear, Q8:No | 2/8 |  |
| Vascular | Robinson et al., 2017 | JBI Cross-sectional | Q1:Yes, Q2:Yes, Q3:Yes, Q4:Yes, Q5:Yes, Q6:Yes, Q7:Yes, Q8:Yes | 8/8 |  |
| Ortho-Oncologic | Thomas et al., 2022 | JBI Cross-sectional | Q1:Yes, Q2:Yes, Q3:Yes, Q4:Yes, Q5:Yes, Q6:Yes, Q7:Yes, Q8:Yes | 8/8 |  |
| Vascular | Goka, 2017 | ROBIS | Phase 1:YES, D1:LOW, D2:LOW, D3:LOW, D4:LOW, Phase 3:YES | LOW RISK | Review aligns with target question on hospital/surgeon characteristics affecting limb outcomes. Clear PICOS framework with pre-specified criteria. Protocol registered on PROSPERO. Comprehensive search strategy. Narrative synthesis appropriately chosen given heterogeneity. |
| Orthoplastic | Joosten, 2024 | ROBIS | Phase 1:YES, D1:LOW, D2:LOW, D3:LOW, D4:LOW, Phase 3:YES | LOW RISK | Primary aim aligns with target question on clinical outcomes and hospital factors. Clear inclusion/exclusion criteria for open tibial fractures. Comprehensive search with medical information specialist. Random-effects meta-analysis appropriate given heterogeneity. |
| Vascular | Nickinson, 2020 | ROBIS | Phase 1:YES, D1:LOW, D2:LOW, D3:LOW, D4:LOW, Phase 3:YES | LOW RISK | Review question on vascular limb salvage services aligns fully with target question. Clear inclusion/exclusion criteria based on GVG definition. Protocol registered on PROSPERO. Comprehensive search across 5 databases. |
| No Center | Yeung, 2021 | ROBIS | Phase 1:YES, D1:LOW, D2:LOW, D3:LOW, D4:LOW, Phase 3:YES | LOW RISK | Systematic review addresses barriers to accessing specialized limb reconstruction services. Clear inclusion/exclusion criteria following Arksey and O'Malley framework. Comprehensive search strategy with systematic screening. Thematic analysis appropriate for scoping methodology. |
| Orthoplastic | Abu El Hawa et al., 2022 | JBI Cohort | D1:Yes, D2:Yes, D3:Yes, D4:Yes, D5:No, D6:Yes, D7:Yes, D8:Unclear, D9:Unclear, D10:No, D11:Yes | 7/11 |  |
| Orthoplastic | Abu El Hawa et al.(2), 2022 | JBI Cohort | D1:Yes, D2:Yes, D3:Yes, D4:Yes, D5:No, D6:Yes, D7:Yes, D8:Unclear, D9:Unclear, D10:No, D11:Yes | 7/11 |  |
| Vascular | Armstrong et al., 2012 | JBI Cohort | D1:Yes, D2:Yes, D3:Yes, D4:No, D5:No, D6:Yes, D7:Yes, D8:Yes, D9:Yes, D10:NA, D11:Yes | 8/11 |  |
| Orthoplastic | Chummun et al., 2013 | JBI Cohort | D1:Yes, D2:Yes, D3:Yes, D4:Unclear, D5:No, D6:Yes, D7:Yes, D8:Unclear, D9:Unclear, D10:Unclear, D11:Yes | 6/11 |  |
| Vascular | Ge et al., 2024 | JBI Cohort | D1:Yes, D2:Yes, D3:Unclear, D4:Unclear, D5:Yes, D6:Unclear, D7:Yes, D8:Yes, D9:Unclear, D10:Unclear, D11:Yes | 6/11 |  |
| Vascular | Houghton et al., 2019 | JBI Cohort | D1:No, D2:No, D3:Unclear, D4:Yes, D5:Yes, D6:Yes, D7:Yes, D8:Yes, D9:Unclear, D10:Unclear, D11:Yes | 6/11 |  |
| Orthoplastic | Seitz et al., 2017 | JBI Cohort | D1:Yes, D2:Yes, D3:Yes, D4:Unclear, D5:No, D6:Yes, D7:Yes, D8:Yes, D9:No, D10:No, D11:Yes | 7/11 |  |
| Vascular | Williams et al., 2018 | JBI Cohort | D1:Yes, D2:Unclear, D3:Unclear, D4:No, D5:No, D6:Unclear, D7:Yes, D8:Yes, D9:Yes, D10:NA, D11:Yes | 5/11 |  |
| Ortho-Trauma | Franz, 2012 | JBI Case Series | D1:Yes, D2:Yes, D3:Yes, D4:Yes, D5:Yes, D6:Yes, D7:Yes, D8:Yes, D9:Yes, D10:Yes | 10/10 |  |
| Ortho-Oncologic | Haidar, 2008 | JBI Case Series | D1:Yes, D2:Yes, D3:Yes, D4:No, D5:No, D6:Yes, D7:Yes, D8:Yes, D9:Yes, D10:Unclear | 7/10 |  |
| Orthoplastic | Strong, 2022 | JBI Case Series | D1:Yes, D2:Unclear, D3:Unclear, D4:Yes, D5:Unclear, D6:Yes, D7:Yes, D8:Yes, D9:Yes, D10:No | 6/10 |  |
| Orthoplastic | Yamamoto, 2022 | JBI Case Series | D1:No, D2:No, D3:No, D4:Unclear, D5:No, D6:Unclear, D7:Unclear, D8:No, D9:Yes, D10:No | 1/10 |  |
| Vascular | O'Banion & Campat, 2024 | JBI Text and Opinion | Q1:Yes, Q2:Yes, Q3:Yes, Q4:Yes, Q5:Yes, Q6:Unclear | 5/6 |  |

**Assessment Tool Definitions and Scoring Systems:**

**MINORS (Methodological Index for Non-Randomized Studies):**

- Non-comparative studies: D1-D8 (maximum 16 points)
- Comparative studies: D1-D12 (maximum 24 points)
- Scoring: 0-2 points per domain (simplified to 3-point scale)
- Domains: D1: A clearly stated aim; D2: Inclusion of consecutive patients; D3: Prospective collection of data; D4: Endpoints appropriate to the aim of the study; D5: Unbiased assessment of the study endpoint; D6: Follow-up period appropriate to the aim of the study; D7: Loss to follow up less than 5%; D8: Prospective calculation of the study size; D9: An adequate control group; D10: Contemporary groups; D11: Baseline equivalence of groups; D12: Adequate statistical analyses

**SANRA (Scale for Assessment of Narrative Review Articles):**

- Maximum score: 12 points (I1-I6, each 0-2 points)
- Scoring guide: 0 points = domain not addressed; 1 point = domain partially addressed; 2 points = domain fully addressed
- Domains: I1 (Justification of importance): evaluates whether the review clearly justifies the importance of the topic; I2 (Statement of aims or questions): assesses whether the review states concrete aims or research questions; I3 (Literature search description): checks if the review provides a description of the literature search methodology; I4 (Referencing): evaluates the adequacy and comprehensiveness of referencing in the review; I5 (Scientific reasoning): assesses the logical flow and scientific reasoning used in presenting the narrative; I6 (Data presentation): evaluates how well the data and evidence are presented to support the review's conclusions

**JBI (Joanna Briggs Institute) Cross-Sectional Studies:**

- Scoring: Yes/No/Unclear
- Domains: Q1: Clearly defined inclusion criteria; Q2: Detailed description of study subjects and setting; Q3: Valid and reliable exposure measurement; Q4: Objective standard criteria for condition measurement; Q5: Identification of confounding factors; Q6: Stated strategies for dealing with confounding factors; Q7: Valid and reliable outcome measurement; Q8: Appropriate statistical analysis

**JBI Cohort Studies:**

- Scoring: Yes/No/Unclear/NA
- Domains: D1: Similarity of groups and recruitment from same population; D2: Similar exposure measurement methods; D3: Valid and reliable exposure measurement; D4: Identification of confounding factors; D5: Strategies to address confounding; D6: Participants free of outcome at study start; D7: Valid and reliable outcome measurement; D8: Adequate follow-up duration; D9: Complete follow-up with reasons for losses described; D10: Strategies to address incomplete follow-up; D11: Appropriate statistical analysis

**JBI Case Series:**

- Scoring: Yes/No/Unclear
- Domains: D1: Clearly stated inclusion criteria; D2: Condition was measured in standard reliable way; D3: Valid methods used for identification of condition; D4: Consecutive inclusion of patients; D5: Complete inclusion of participants; D6: Clear reporting of demographics; D7: Clear reporting of clinical information; D8: Clear reporting of outcomes of follow-up results; D9: Clear reporting of presenting site clinics demographic information; D10: Appropriate statistical analysis

**JBI Text and Opinion:**

- Scoring: Yes/No/Unclear
- Domains: Q1: Source of opinion clearly identified; Q2: Source has standing in field of expertise; Q3: Interests of relevant population are central focus; Q4: Stated position results from analytical process with logical opinion; Q5: Reference to extant literature; Q6: Incongruence with literature/sources logically defended

**ROBIS (Risk of Bias in Systematic Reviews):**

- Risk levels: LOW, MODERATE, HIGH
- Assessment phases: Phase 1 (Assessing Relevance): evaluates whether the systematic review addresses the specific research question clearly and appropriately; Domain 1 (Study Eligibility Criteria): assesses the clarity and appropriateness of the eligibility criteria for study selection; Domain 2 (Identification and Selection of Studies): evaluates the comprehensiveness of the search strategy and methods used to identify studies; Domain 3 (Data Collection and Study Appraisal): looks at the systematic approach to data collection, risk of bias assessment in individual studies, and use of appropriate quality assessment tools; Domain 4 (Synthesis and Findings): evaluates whether the data were synthesized appropriately, with attention to study heterogeneity and sensitivity analyses; Phase 3 (Judging Risk of Bias): evaluates the overall risk of bias, considering how concerns were addressed and whether the conclusions were appropriately balanced
